# β_2_-Adrenergic receptor modulates mitochondrial metabolism and disease progression in recurrent/metastatic HPV(+) HNSCC

**DOI:** 10.1038/s41389-018-0090-2

**Published:** 2018-10-08

**Authors:** Christopher T. Lucido, Juan L. Callejas-Valera, Paul L. Colbert, Daniel W. Vermeer, W. Keith Miskimins, William C. Spanos, Paola D. Vermeer

**Affiliations:** Cancer Biology and Immunotherapies Group, Sanford Research, 2301 East 60th St North, Sioux Falls, SD 57104 USA

## Abstract

The incidence of human papillomavirus-associated head and neck squamous cell carcinoma (HPV[ + ] HNSCC) is rapidly increasing. Although clinical management of primary HPV( + ) HNSCC is relatively successful, disease progression, including recurrence and metastasis, is often fatal. Moreover, patients with progressive disease face limited treatment options and significant treatment-associated morbidity. These clinical data highlight the need to identify targetable mechanisms that drive disease progression in HPV( + ) HNSCC to prevent and/or treat progressive disease. Interestingly, β-adrenergic signaling has recently been associated with pro-tumor processes in several disease types. Here we show that an aggressive murine model of recurrent/metastatic HPV( + ) HNSCC upregulates β_2_-adrenergic receptor (β2AR) expression, concordant with significantly heightened mitochondrial metabolism, as compared with the parental model from which it spontaneously derived. β-Adrenergic blockade effectively inhibits in vitro proliferation and migratory capacity in this model, effects associated with an attenuation of hyperactive mitochondrial respiration. Importantly, propranolol, a clinically available nonselective β-blocker, significantly slows primary tumor growth, inhibits metastatic development, and shows additive benefit alongside standard-of-care modalities in vivo. Further, via CRISPR/Cas9 technology, we show that the hyperactive mitochondrial metabolic profile and aggressive in vivo phenotype of this recurrent/metastatic model are dependent on β2AR expression. These data implicate β2AR as a modulator of mitochondrial metabolism and disease progression in HPV( + ) HNSCC, and warrant further investigation into the use of β-blockers as low cost, relatively tolerable, complementary treatment options in the clinical management of this disease.

## Introduction

Annually, over 500,000 patients are diagnosed with head and neck squamous cell carcinoma (HNSCC) worldwide^1^. Human papillomavirus (HPV) infection is implicated in ~25% of all HNSCC cases (HPV( + ) HNSCC)^2^. The incidence of this distinct subtype has increased by over 200% in recent decades^1^ and may soon exceed the disease burden of cervical cancer in some developed countries^3^. Although HPV( + ) tumors typically respond more favorably to standard-of-care chemoradiation therapy (CRT) than their HPV( − ) counterparts, progressive disease remains a significant problem^4^. Patients that experience disease progression, including metastasis and/or recurrence, face exceedingly poor prognoses, limited treatment options, and significant treatment-associated morbidity^5^. These clinical data emphasize the need to identify cellular mechanisms that contribute to the development of recurrent/metastatic HPV( + ) HNSCC. Understanding these mechanisms can translate to the development of novel, well-tolerated therapeutic interventions.

Immune escape, therapeutic resistance, and enhanced metastatic capacity are among the many factors known to contribute to treatment failure and progressive disease^6,7^. A growing body of evidence now suggests that adrenergic signaling also modulates disease progression^8,9^. β-Adrenergic signaling, in particular, has been shown to control cellular processes known to contribute to tumor initiation, progression, and metastasis^8–10^. Several studies have documented β-adrenergic receptor expression in various tumor types and have demonstrated that signaling through these receptors may contribute to tumor cell proliferation, migration, and invasion^11–18^. These effects are primarily mediated by β_2_-adrenergic receptor (β2AR) and are susceptible to inhibition by β-adrenergic antagonists (β-blockers)^11–18^. In HNSCC, a study by Shang et al.^15^ found that β2AR expression was significantly more common in oral squamous cell carcinoma than normal oral mucosa, and that β2AR positivity was associated with tumor size, stage, and lymph node metastasis. Retrospective clinical analyses have demonstrated improved outcomes in breast and ovarian cancer patients taking β-blockers during the course of their disease, an effect dependent on the use of nonselective β-blockers (i.e., those that block β_1_ and β_2_ receptors, as opposed to β_1_-selective agents)^19,20^. A recent clinical trial in the setting of thick cutaneous melanoma found that adjuvant propranolol (a nonselective β-blocker) significantly improved progression-free survival^21^. Given that they are already Food and Drug Administration (FDA)-approved, low cost, and generally well-tolerated, β-blockers have the potential to move swiftly from the bench to the bedside as complementary anti-cancer agents.

Here, we identify upregulated β2AR expression, concordant with hyperactive mitochondrial metabolism, in an aggressive, treatment-resistant, recurrent/metastatic murine model of HPV( + ) HNSCC as compared with the parental model from which it spontaneously derived. Although not well understood, β-adrenergic receptor activity has been shown to modulate mitochondrial metabolism in several studies^22–24^ and increased mitochondrial activity has been associated with aggressive disease^25–27^. We thus hypothesized that β2AR contributes to an aggressive phenotype in HPV( + ) HNSCC, in part through modulation of mitochondrial activity, and may be a worthwhile therapeutic target in this setting. Here, we show that β-adrenergic blockade displays significant anti-proliferative and anti-migratory activity in vitro and is associated with attenuation of mitochondrial respiration in this recurrent/metastatic model. These effects translate to slower tumor growth and decreased metastatic development in vivo in mice treated with propranolol. We also show that propranolol displays additive benefit alongside current standard-of-care therapies. Using CRISPR/Cas9 technology, we identify β2AR expression as critical to this model’s aggressive in vivo growth phenotype and hyperactive mitochondrial respiration profile. These data implicate β2AR in the modulation of mitochondrial metabolic activity and disease progression in HPV( + ) HNSCC, and identify β2AR as a potential therapeutic target, particularly in the recurrent/metastatic setting where effective treatment options are limited.

## Results

### **A highly aggressive recurrent/metastatic model of HPV(** **+** **) HNSCC upregulates β2AR expression**

**Fig. 1:**
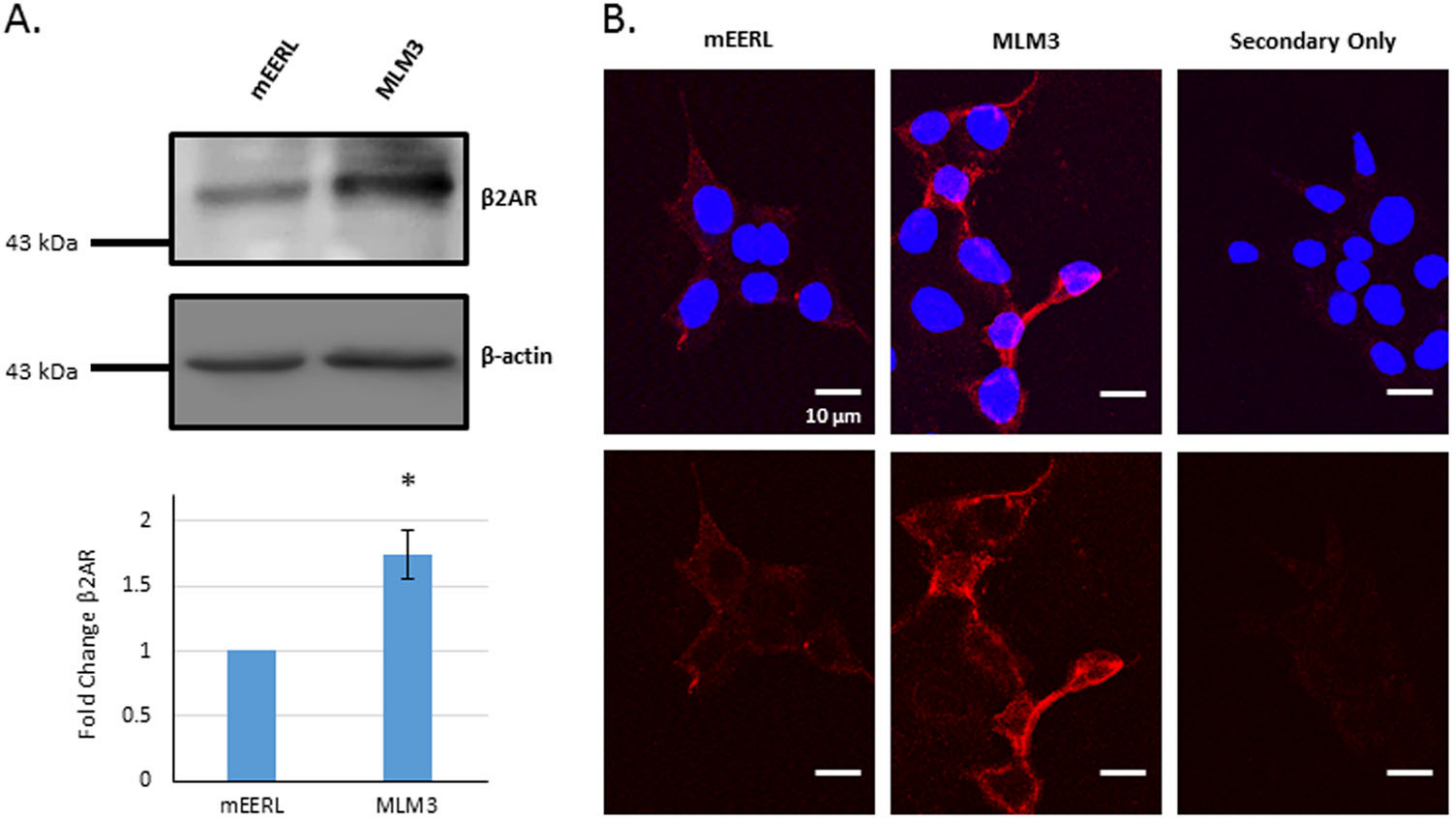
β2AR expression in mEERL and its recurrent/metastatic derivative, MLM3. **a** Western blot analysis of β2AR expression in MLM3 and mEERL cell lines (top), and relative expression data (bottom), confirming upregulation in the recurrent/metastatic MLM3 model, as compared with the parental mEERL model from which it spontaneously derived (columns represent mean fold change in β2AR protein expression [normalized to β-actin loading control] ± SEM from *n* = 4 independent biological replicates; p-value reflects results of Mann–Whitney test). **b** Unfixed, unpermeabilized immunostaining of β2AR (red) on mEERL (left) and MLM3 (middle) cells showing membrane localization of the receptor (secondary antibody-only staining is shown in the right panel). Nuclei were stained with DAPI (blue). Top: overlay; bottom: β2AR only

Our laboratory previously generated and characterized a murine model of HPV( + ) HNSCC derived from mouse oropharyngeal epithelial cells stably expressing HPV16 E6 and E7 oncoproteins, H-Ras, and luciferase (mEERL)^28–31^. More recently, our laboratory characterized several clonal cell lines derived from lung metastases that spontaneously developed following CRT failure in a mouse implanted with a mEERL tumor (mEERL lung metastasis cell lines, MLMs)^32^. Importantly, MLM tumors are significantly more aggressive than parental mEERL tumors in terms of primary tumor growth, metastatic development, and treatment resistance^32^, making them a useful model system to study translational therapeutic strategies for progressive HPV( + ) HNSCC. To identify potential molecular contributors to disease progression in HPV( + ) HNSCC, microarray analysis (published in Vermeer et al.^32^; GEO accession #GSE68935) was used to compare gene expression profiles of the recurrent/metastatic MLM cell lines with that of the parental mEERL cells from which they spontaneously derived. Interestingly, the MLM cell lines significantly upregulate β2AR expression as compared with the parental mEERL cells (^32^ GEO accession #GSE68935). These data suggest that this receptor may critically contribute to progressive HPV( + ) HNSCC. As MLM3 is the fastest growing, most metastatic, and most treatment-resistant clone, it was the focus of our studies. Consistent with microarray data, MLM3 displays upregulated β2AR expression at the protein level (Fig. 1a). Unfixed, unpermeabilized immunostaining of β2AR identified membrane localization of this receptor in both the parental mEERL and the metastatic MLM3 cells (Fig. 1b).

### β-Adrenergic blockade displays anti-tumor activity in murine and human HNSCC cell lines

**Fig. 2:**
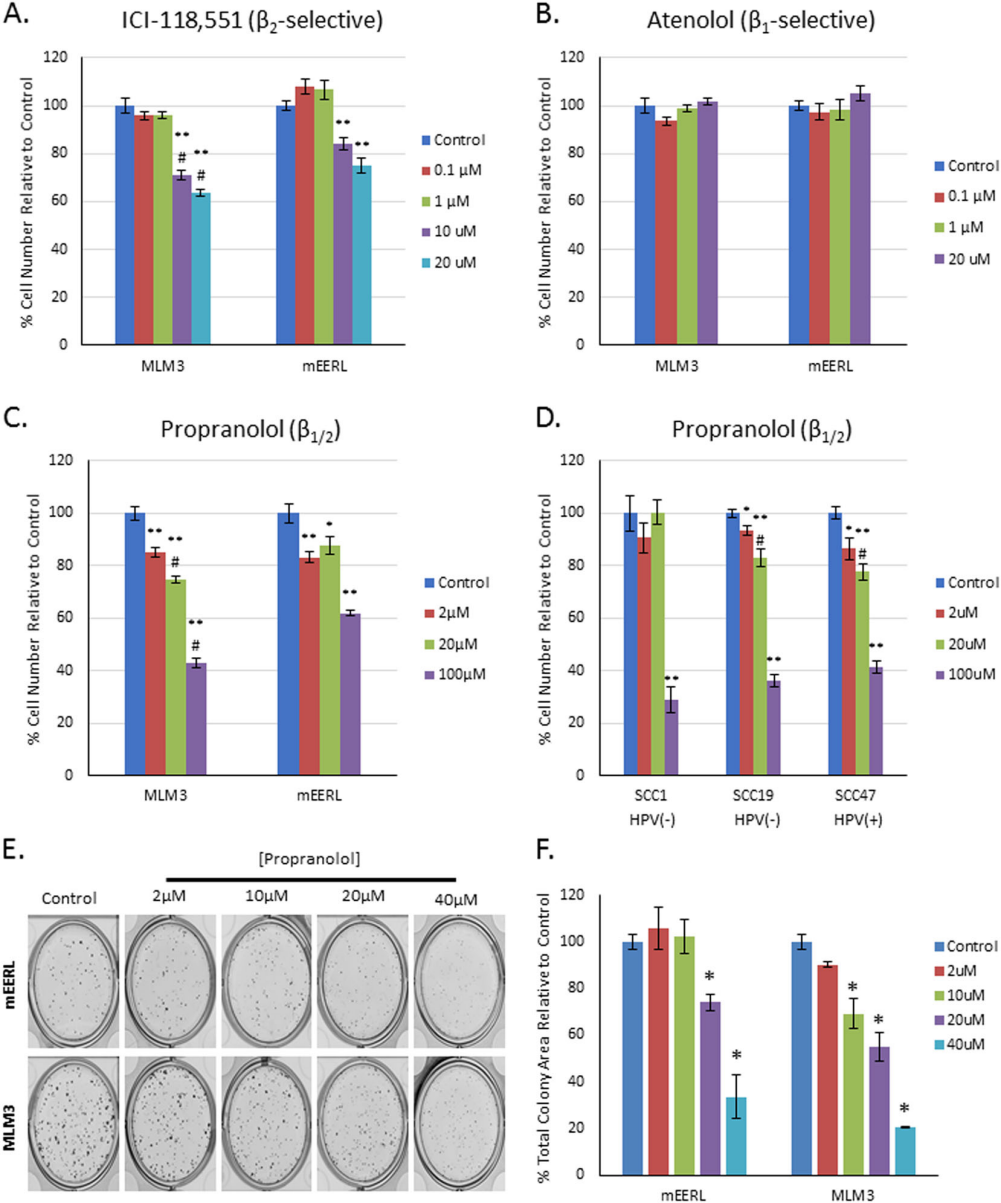
β2AR blockade displays anti-tumor activity in murine and human HNSCC cell lines. MTT assay results of MLM3 or mEERL cells treated for 48 h with the indicated concentrations of the β2-selective blocker, ICI-118,551 (**a**), the β1-selective blocker, atenolol (**b**), or the nonselective β-blocker, propranolol (**c**), indicating decreased cell number with β2AR blockade (*p < 0.02 compared with control; ** *p* ≤ 0.004 compared with control; #*p* ≤ 0.01 compared with mEERL cells at identical drug concentration). Experiments were repeated twice with similar results. Columns represent mean ± SEM from *n* = 6 independent biological replicates; *p*-value reflects results of Mann–Whitney test. **d** Human HPV( + ) and HPV( − ) HNSCC cell lines are sensitive to the tumor effects of propranolol (*p < 0.03 compared with control; **p ≤ 0.004 compared with control; #*p* ≤ 0.03 compared with SCC1 cells at identical drug concentration). Columns represent mean ± SEM from *n* = 6 independent biological replicates; *p*-value reflects results of Mann–Whitney test. **e** Representative images and (**f**) results of a colony-forming assay on mEERL or MLM3 cells treated with increasing doses of propranolol (*p < 0.03 compared with control). Columns represent mean ± SEM from *n* = 4 independent biological replicates; *p*-value reflects results of Mann–Whitney test

To investigate the effects of β-blockers in our HPV( + ) HNSCC tumor models, we conducted a series of MTT (3-(4,5-dimethylthiazol-2-yl)-2,5-diphenyltetrazolium bromide) assays. mEERL or MLM3 cells were treated with drugs selective for either β1AR (atenolol) or β2AR (ICI-118,551) for 48 h. ICI-118,551 significantly decreased both MLM3 and mEERL cell number (Fig. 2a), with the MLM3s displaying greater sensitivity to this effect. Atenolol did not significantly affect cell number in either cell line (Fig. 2b), with the exception of 100 μM, which induced a slight decrease in cell number, likely related to loss of selectivity (Supplemental Fig. 1A). As β2AR-selective antagonists are not clinically available, we sought to determine whether a clinically available agent could induce similar effects. Thus, we evaluated propranolol, a nonselective β-blocker. As with ICI-118,551, propranolol decreased the number of both mEERL and MLM3 cells, with the MLM3s again more sensitive to this effect (Fig. 2c and Supplemental Fig. 1B). To investigate whether this effect is conserved in human HNSCC cells, and to determine whether this response is dependent on HPV status, three human HNSCC cell lines (2 HPV( − ): SCC1/19; 1 HPV( + ): SCC47) were treated with atenolol, ICI-118,551, or propranolol for 48 h. As with the mEERL and MLM3 cell lines, atenolol only induced a slight decrease in SCC47 cell number (Supplemental Fig. 1C), whereas ICI-118,551 and propranolol significantly decreased cell number in all three SCC cell lines (Fig. 2D and Supplemental Fig. 1C), indicating that this effect is not limited to our murine models, nor limited to HPV( + ) HNSCC.

To further support the decrease in cell number observed via MTT assay, mEERL, and MLM3 colony formation was assessed in response to increasing doses of propranolol. Similar to the MTT assay, propranolol significantly decreased total colony area of both mEERL and MLM3 cells (Fig. 2e, f).

Canonically, β-adrenergic signaling leads to an accumulation of cyclic AMP (cAMP), which subsequently activates protein kinase A (PKA). To investigate whether propranolol’s anti-tumor effects are related to blockade of canonical β-adrenergic signaling, we assessed the levels of phosphorylated PKA substrate in response to propranolol (Supplemental Fig. 1D). Interestingly, doses required to achieve anti-tumor activity in the MTT and colony-forming assays (Fig. 2c, f, respectively) were lower than those required to decrease the levels of phosphorylated PKA substrate. Thus, propranolol’s anti-tumor activity in these models appears to be independent of cAMP/PKA inhibition.

### Propranolol acts in a cytostatic manner on MLM3 and mEERL cells

**Fig. 3:**
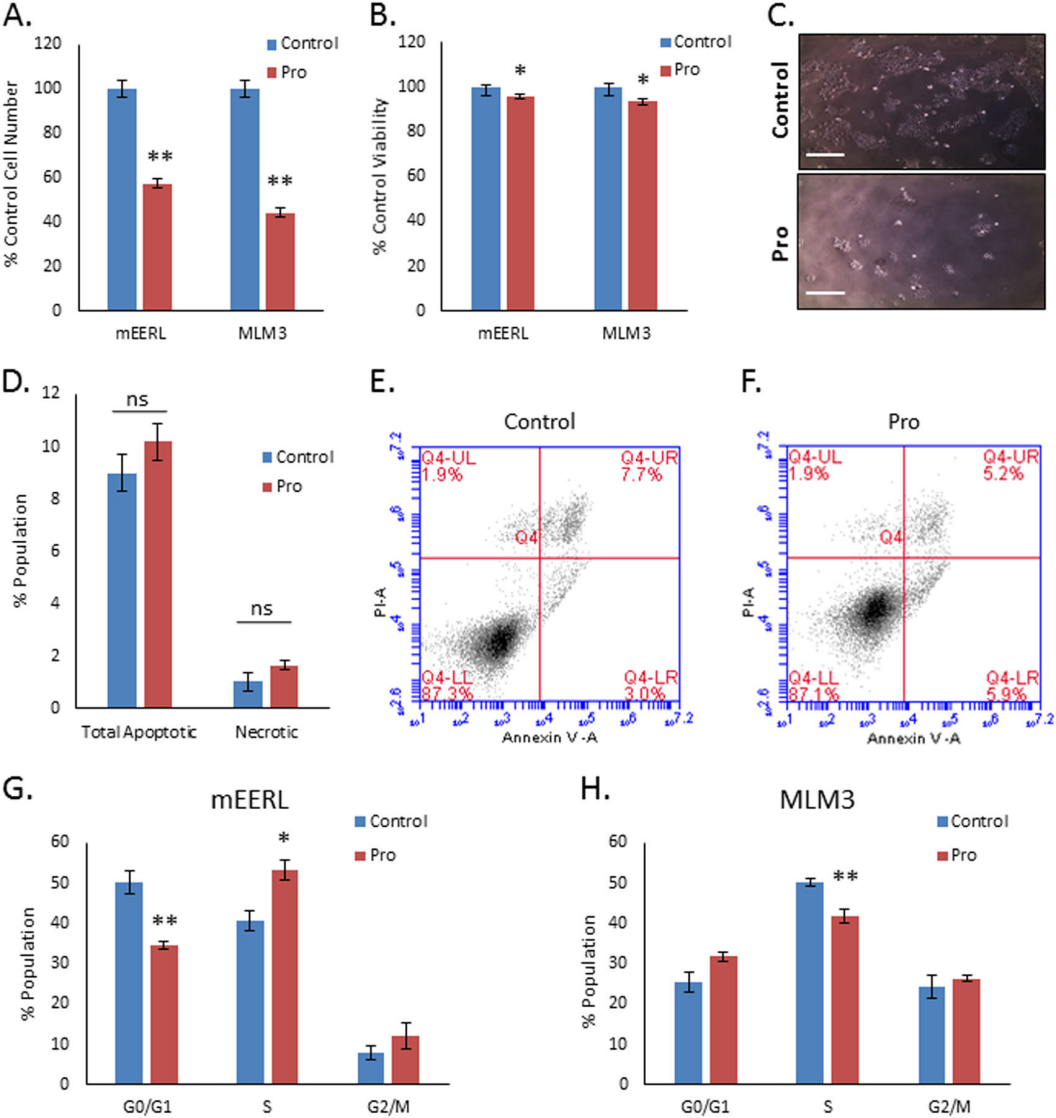
Propranolol acts in a cytostatic manner on MLM3 and mEERL cells. **a** MTT assay results were confirmed by live cell counting after treating mEERL (left) or MLM3 (right) cells for 48 h with 40 μM propranolol (**p ≤ 0.001 compared with control). Columns represent mean ± SEM from independent biological replicates; *n* = 8 [control] or 7 [pro]; results from two independent experiments were normalized to untreated control and pooled; *p*-value reflects results of Mann–Whitney test. **b** Trypan blue exclusion staining of mEERL (left) or MLM3 (right) cells treated with 40 μM propranolol for 48 h reveals propranolol’s effects on cell viability. Results indicate a small (but statistically significant) degree of cell death in both cell lines following 48 h propranolol treatment (*p < 0.05 compared with control). Columns represent mean ± SEM from independent biological replicates; *n* = 8 [control] or 7 [pro]; results from two independent experiments were normalized to untreated control and pooled; *p*-value reflects results of Mann–Whitney test. **c** Representative bright-field images of untreated (control, top) or propranolol treated (Pro, bottom) MLM3 cells. Images were taken at × 4 magnification. **d** Results and **e**, **f** representative scatter plots of annexin V/PI cell death assay following 48 h treatment of MLM3 cells with 40 μM propranolol, indicating that propranolol does not significantly induce apoptosis or necrosis (total apoptotic = % annexin V positive; total necrotic = % PI positive, annexin V negative). Columns represent mean ± SEM from *n* = 4 independent biological replicates, represented by data points (ns = not significant). **g**, **h** Results of PI cell cycle analysis indicating changes in cell cycle distribution in mEERL (**g**) (*n* = 4 [control] or 3 [pro]) and MLM3 cells (**h**) (*n* = 4) following 48 h treatment with 40 μM propranolol (*p < 0.05 compared with control, **p ≤ 0.007 compared with control). Columns represent mean ± SEM from independent biological replicates; *p*-value reflects results of unpaired *t*-test

MTT assays cannot definitively determine whether decreased cell number is due to decreased cellular proliferation or increased cell death. Thus, to investigate the cause of decreased cell number in response to propranolol, MLM3 or mEERL cells were treated with 40 µM propranolol (approximate concentration necessary to achieve 50% of control cell number in MLM3s with MTT assay) for 48 h, stained with trypan blue to identify dead cells, and counted. As seen in the MTT assays, propranolol significantly decreased cell number (Fig. 3a). However, the viability of propranolol-treated cells remained within 6% of control for both cell types (Fig. 3b), suggesting that attenuated proliferation, rather than cytotoxicity, is primarily responsible for the decrease in cell number during propranolol treatment. Furthermore, flow cytometric analysis of apoptosis and necrosis via annexin V/propidium iodide (PI) staining indicated no significant differences between control and propranolol-treated cells (Fig. 3d, e, f), whereas cell cycle analysis indicated perturbations in the cell cycle distribution of both mEERL and MLM3 cell lines (Fig. 3g, h; Supplemental Fig. 2A and 2B). Taken together, these data indicate that propranolol acts in a cytostatic manner. Of note, propranolol did not significantly inhibit the proliferation of normal tonsil epithelium (Supplemental Fig. 1E), suggesting the effect may be relatively selective for tumor cells.

### **Propranolol inhibits MLM3 migratory capacity**

**Fig. 4:**
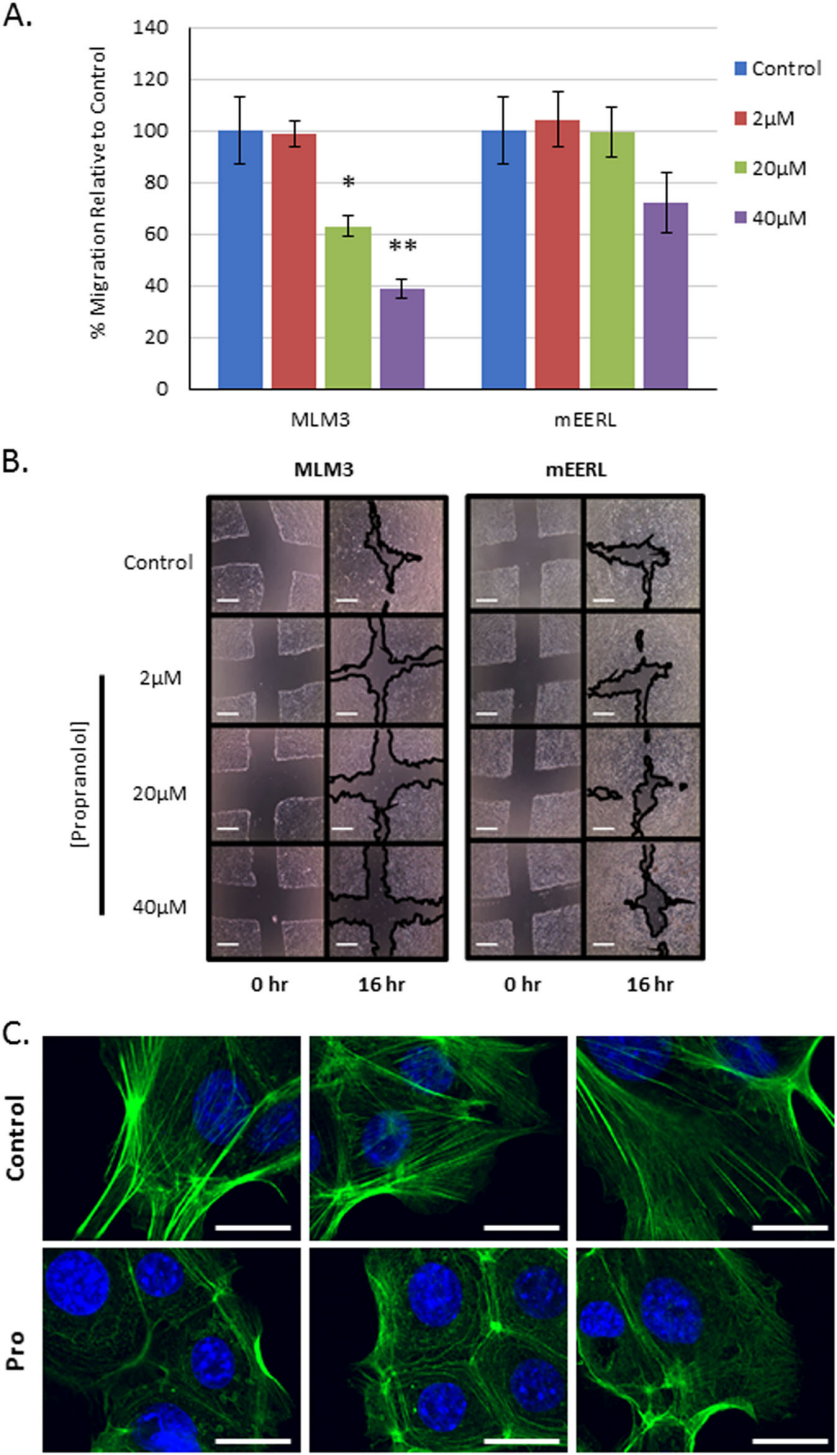
Propranolol inhibits MLM3 migratory capacity. **a** Results of a scratch wound migration assay, in which MLM3 (left) or mEERL (right) cells were treated with the indicated concentrations of propranolol for 16 h, indicating that propranolol significantly inhibits the migration of the highly metastatic MLM3 cell line (*p* = 0.004 and 0.002 at 20 μM and 40 μM, respectively), whereas the mEERL cell line is relatively unaffected (*p* = 0.18 at 40 μM). Experiment was repeated twice with similar results. Columns represent mean ± SEM from *n* = 6 (MLM3) or 5 (mEERL) independent biological replicates; *p*-value reflects results of Mann–Whitney test. **b** Representative bright-field images of MLM3 cells (left) or mEERL cells (right) immediately following wound generation (0 h) and after 16 h of propranolol treatment (16 h). Images were taken at × 4 magnification. Scale bar = 1 mm. **c** MLM3 cells were treated with propranolol for 16 h and stained with phalloidin to label F-actin (green). Nuclei were stained with DAPI (blue). Scale bar = 10μm

β-Adrenergic signaling has been shown to modulate various cytoskeletal remodeling processes known to regulate cellular migration^9^. As such, β-adrenergic blockade may represent an intriguing strategy to limit a cell’s migratory capacity and impede its ability to metastasize. We sought to investigate whether propranolol displays anti-migratory activity in MLM3 or mEERL cells using a scratch wound migration assay. Propranolol significantly attenuated the migratory capacity of MLM3 cells (Fig. 4a left and 4b left), which are highly metastatic in vivo^32^. Further supporting an anti-migratory role for propranolol, phalloidin staining revealed striking defects in actin polymerization in MLM3 cells in response to treatment (Fig. 4c). Interestingly, mEERL cells, which are inherently much less metastatic than MLM3s^32^, maintained their migratory capacity (Fig. 4a right and 4b right), displaying only a nonsignificant decrease in migration at the highest propranolol dose used, suggesting that mEERL cells may not significantly rely on β-adrenergic signaling for motility.

### **Propranolol attenuates MLM3 hyperactive mitochondrial metabolic activity**

**Fig. 5:**
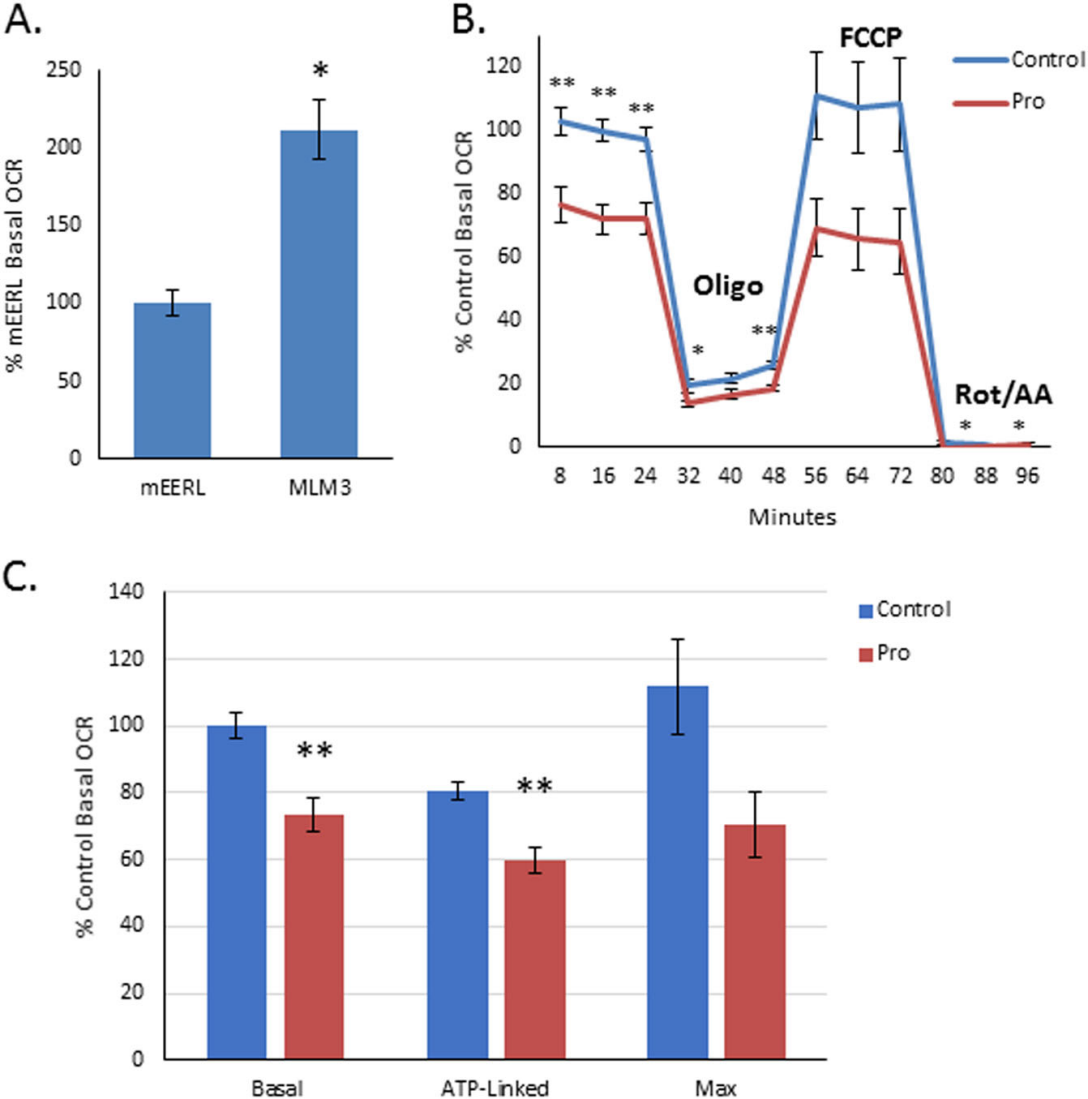
Propranolol attenuates MLM3 hyperactive mitochondrial metabolism. **a** Seahorse assay results comparing OCR in mEERL cells (left) and MLM3 cells (right), indicating significantly increased mitochondrial metabolic activity in the recurrent/metastatic MLM3 cell line (*p < 0.001). Columns represent mean ± SEM from independent biological replicates; *n* = 10 [mEERL] or 9 [MLM3]; results from two independent experiments were pooled; *p*-value reflects results of Mann–Whitney test; all values were normalized to total protein, adjusted for non-mitochondrial respiration, and normalized to % mEERL basal OCR. **b**, **c** Seahorse assay results showing the effects of propranolol on MLM3 mitochondrial metabolism, indicating that propranolol significantly attenuates MLM3 basal (average of three OCR readings before oligo injection) and ATP-linked OCR (difference between basal OCR and OCR following oligo injection). Maximal OCR (highest OCR following FCCP injection) was also attenuated by propranolol, though this finding was shy of statistical significance (*p < 0.05; **p ≤ 0.006). Columns and data points represent mean ± SEM from *n* = 9 independent biological replicates; results from two independent experiments were pooled; *p*-value reflects results of Mann–Whitney test; all values were normalized to total protein, adjusted for non-mitochondrial respiration, and normalized to % untreated control basal OCR

Mitochondrial metabolism has proven critical to tumorigenesis^26,33^ and increased mitochondrial activity has been associated with progressive disease and treatment resistance^25–27^. Moreover, β2AR activity has been shown to modulate mitochondrial metabolism in several cell types^22–24^. Thus, we sought to compare mitochondrial metabolism in the mEERL and MLM3 cell lines, and to investigate the metabolic effects of β-adrenergic blockade. Using a Seahorse XF analyzer to measure oxygen consumption rate (OCR), we found that the MLM3s exhibited a twofold increase in mitochondrial respiration relative to the parental mEERL cells (Fig. 5a), suggesting that hyperactive mitochondrial metabolism may contribute to an aggressive tumor phenotype in HPV( + ) HNSCC.

To investigate the effect of β-adrenergic blockade on mitochondrial metabolism, MLM3 cells were treated with propranolol for 24 h and basal mitochondrial respiration was measured before sequential injections of: (1) oligomycin (oligo) to inhibit ATP synthase and allow for measurement of ATP-linked respiration; (2) the uncoupling agent, carbonyl cyanide-4-(trifluoromethoxy)phenylhydrazone (FCCP) to drive maximal respiration and allow for measurement of maximal OCR; and (3) a mixture of rotenone and antimycin A (rot/AA) to shut down the electron transport chain and allow for measurement of non-mitochondrial respiration. Propranolol significantly attenuated MLM3 basal (*p* = 0.006) and ATP-linked OCR (*p* = 0.005), and decreased maximal respiration (*p* = 0.077; Fig. 5b, c). Thus, β-adrenergic antagonism is capable of modulating the hyperactive metabolic phenotype of the MLM3s. As proliferation and migration are energetically demanding processes, this effect may explain both the anti-proliferative and anti-migratory activity of β-adrenergic antagonism.

### **Propranolol inhibits MLM3 primary tumor growth and metastatic developmentin vivo**

**Fig. 6:**
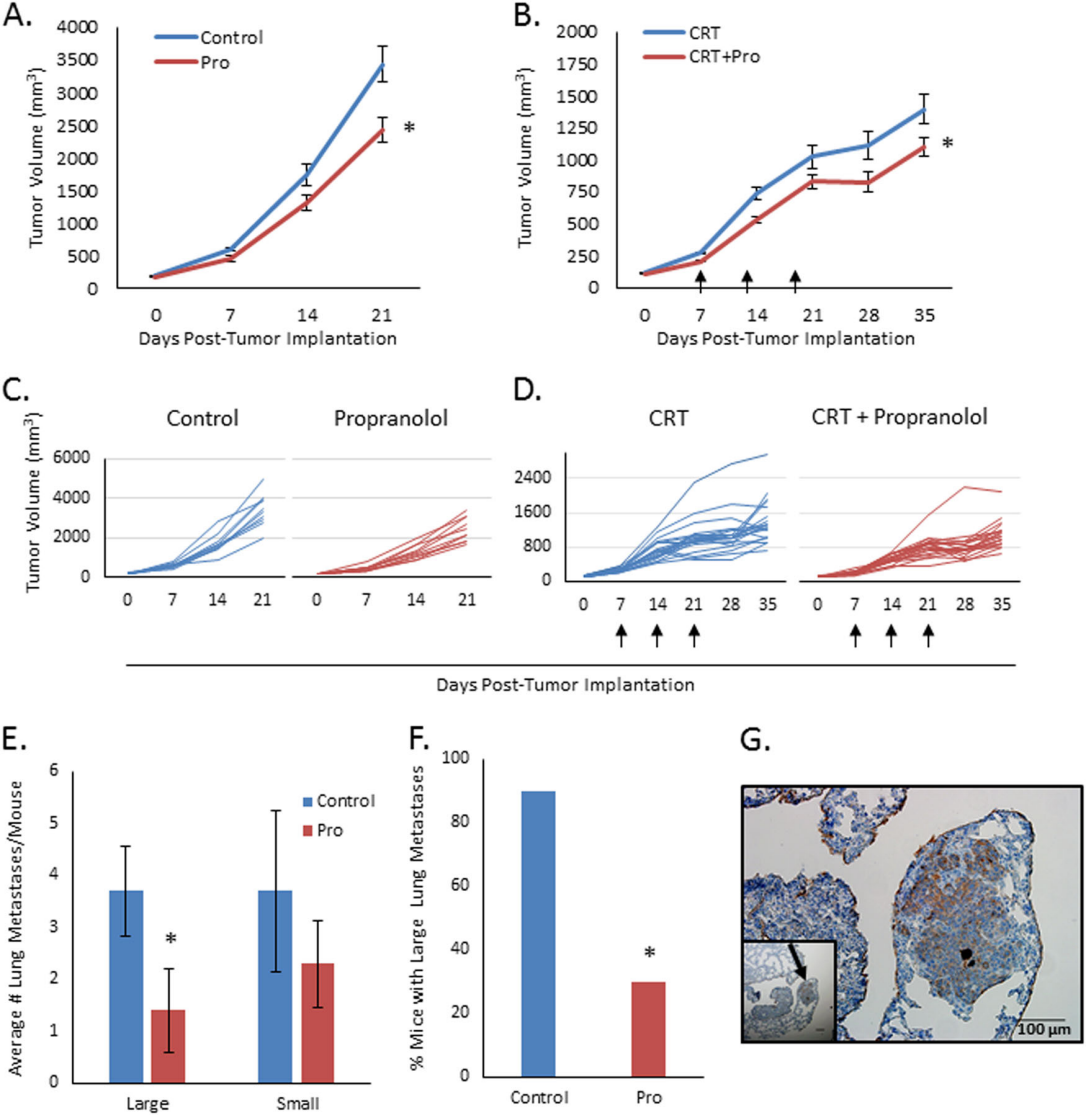
Propranolol significantly inhibits MLM3 primary tumor growth and metastatic development and shows additive benefit alongside standard-of-care therapies. **a** Average MLM3 tumor volume curves for mice treated with 2 mg/kg daily IP propranolol (or vehicle), showing that propranolol significantly inhibits MLM3 primary tumor growth (*p* = 0.007). Data points represent mean ± SEM of *n* = 10 mice; *p*-value reflects results of unpaired, two-tailed student’s t-test. **b** Average MLM3 tumor volume curves for mice treated with 3 weekly rounds of CRT (arrows) plus 2 mg/kg daily IP propranolol (or vehicle), showing that propranolol displays additive benefit alongside standard-of-care therapies in this model (*p* = 0.024). Data points represent mean ± SEM of *n* = 20 mice; data failed Shapiro–Wilk normality testing [*p* < 0.05] and was analyzed by Mann–Whitney test. **c** Individual tumor volume curves for mice treated with vehicle (left) or propranolol (right). **d** Individual tumor volume curves for mice treated with three rounds of CRT (arrows) plus vehicle (left) or propranolol (right). **e** IHC quantification of large ( > 70 µm) or small lung metastases, indicating a significant reduction in the average number of large metastatic lung nodules in propranolol-treated mice versus controls (*p* = 0.031); small metastatic nodules were also decreased, although this finding was not statistically significant. Columns represent mean ± SEM from *n* = 10 mice; three random independent lung sections from each animal were analyzed; data failed Shapiro–Wilk normality testing [*p* < 0.05] and was analyzed by Mann–Whitney test. **f** Number of mice per group displaying large metastatic lung nodules. Significantly fewer propranolol-treated mice displayed IHC evidence of large metastatic nodules, as compared with control animals (*p* = 0.019; *χ*^2^-test). **g** Representative image (× 40 magnification) of a cytokeratin-positive metastatic lung nodule (inset at × 4 magnification)

To investigate whether the in vitro effects of propranolol translate to improved outcomes in vivo, mice were implanted with MLM3 tumors and treated daily with 2 mg/kg propranolol (or vehicle) beginning on the day of implantation. Importantly, body surface area normalization of 2 mg/kg in mice yields n human equivalent dose of < 0.2 mg/kg, which would equate to a low dose of propranolol^34,35^. We found that single-agent propranolol significantly inhibited MLM3 primary tumor growth, leading to approximately a 30% reduction in tumor volume as compared to control animals at study endpoint (Fig. 6a, c). Interestingly, the slower growing mEERL tumors did not show a response to propranolol at this dose (Supplemental Fig. 3), suggesting that MLM3s may be more dependent on β2AR to maintain their phenotype. Alternatively, given that a higher dose of β-blockers was required to achieve similar in vitro effects in the mEERL cells, 2 mg/kg may be below the threshold necessary to achieve in vivo response in this tumor model.

We next sought to investigate whether the addition of propranolol to current standard-of-care CRT displayed any further benefit as compared with CRT alone. Mice were implanted with MLM3 tumors and treated with 2 mg/kg/day intraperitoneal (IP) propranolol (or vehicle) and 3 weekly rounds of cisplatin and radiation starting 1 week post-tumor implantation. The combination of propranolol and CRT showed significant improvement in controlling primary tumor growth as compared to CRT alone (Fig. 6b, d) without added toxicity (as assessed by changes in body weight; Supplemental Fig. 4), indicating that propranolol may display benefit as a therapeutic adjuvant.

Given propranolol’s significant anti-migratory activity in the MLM3s, we next assessed whether propranolol could inhibit the development of lung metastases in mice implanted with these highly metastatic tumors. Mice were treated with 2 mg/kg/day propranolol (or vehicle) and sacrificed simultaneously. Three independent lung sections from each animal were stained for pan-cytokeratin to quantify metastatic nodules. Propranolol significantly decreased the number of large metastatic nodules by over 50% (Fig. 6e left) and led to significantly less mice with immunohistochemical evidence of large metastases (Fig. 6f). The number of small metastatic nodules also decreased with propranolol treatment (Fig. 6e right), although this value was not statistically significant.

### **The hyperactive mitochondrial metabolic profile and aggressivein vivophenotype of MLM3 tumors are dependent on β2AR expression**

**Fig. 7:**
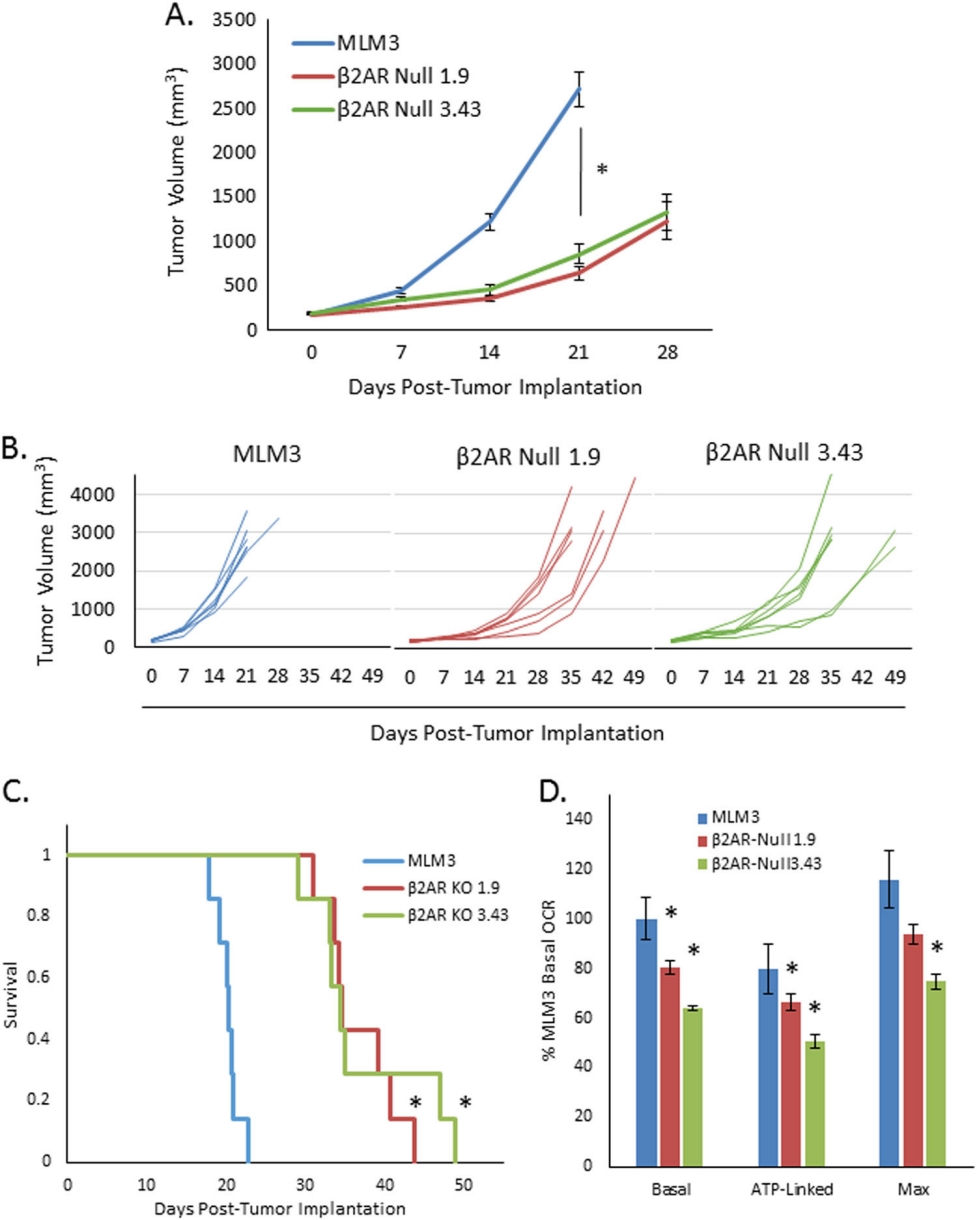
The aggressive in vivo phenotype and hyperactive mitochondrial respiration profile of MLM3 tumors are dependent on their expression of β2AR. **a** Average tumor volume curves for mice implanted with MLM3 or β2AR-null tumors, showing that the aggressive in vivo growth phenotype of MLM3 tumors is dependent β2AR (*p* < 0.001). Data points represent mean ± SEM of *n* = 7 mice; group averages were calculated until the first mouse from each respective group succumbed to disease; *p*-value reflects results of Mann–Whitney test. **b** Individual tumor volume curves for mice implanted with MLM3 (left) or β2AR-null tumors (middle, right). **c** Kaplan–Meier survival curve showing prolonged survival in mice implanted with β2AR-null tumors compared with their MLM3-bearing counterparts (*p* < 0.001; log-rank test). **d** Seahorse assay results showing the effects of β2AR knockout on parameters of MLM3 mitochondrial metabolism. β2AR-null clones display attenuated mitochondrial respiration as compared with the parental MLM3 cell line (*p ≤ 0.03 as compared with MLM3). Columns represent mean ± SEM from *n* = 4 independent biological replicates, represented by data points; *p*-value reflects results of Mann–Whitney test; all values were normalized to total protein and are depicted as a percent relative to MLM3 basal OCR

Although MLM3 tumors upregulate β2AR expression and display significant sensitivity to the anti-tumor activity of β-adrenergic blockade, it remains possible that β2AR alone is not a critical driver of their phenotype. Thus, we utilized CRISPR/Cas9 technology to generate stable β2AR-null clonal cell lines from the MLM3s to assess the necessity of β2AR in the achievement of their aggressive in vivo phenotype. Two clones (generated by two distinct targeting strategies) were selected for assessment of in vivo tumor growth (Supplemental Fig. 2A–C). β2AR-null tumors grew significantly slower than MLM3 tumors (Fig. 7a, b) and mice harboring β2AR-null tumors survived significantly longer than their MLM3-bearing counterparts (Fig. 7c). Furthermore, similar to results seen during propranolol treatment (Fig. 5b, c), loss of β2AR significantly attenuated mitochondrial metabolism (Fig. 7d), suggesting that hyperactive mitochondrial metabolism in these cells is dependent on β2AR expression. These findings are consistent with β2AR having an essential role in driving MLM3 hyperactive mitochondrial metabolism and conferring their aggressive in vivo growth phenotype.

## Discussion

The propensity for β-adrenergic signaling to drive numerous cellular processes that contribute to tumorigenesis and disease progression has previously been demonstrated^8–10^. The data presented here corroborate and extend these findings, implicating β2AR in the modulation of tumor cell metabolism and progressive disease in HPV( + ) HNSCC. Here we show that β2AR expression is upregulated, concordant with heightened mitochondrial metabolic activity, in a highly aggressive, treatment-resistant, syngeneic murine model of recurrent/metastatic HPV( + ) HNSCC as compared with the parental tumor model from which it spontaneously derived. We show that treatment with β-blockers inhibits cellular proliferation and migration, effects which are associated with an attenuation of mitochondrial metabolic activity. Moreover, we show that propranolol inhibits in vivo tumor growth and metastatic development in this model and displays additive benefit alongside standard-of-care CRT, indicating potential benefit as an adjuvant therapy. Further, we show that β2AR expression is required to achieve the aggressive in vivo phenotype of this model and maintain its hyperactive mitochondrial respiration profile.

The data presented here correlate mitochondrial activity with progressive disease in HPV( + ) HNSCC and implicate β2AR as a regulator of this activity. Mitochondrial metabolism is gaining traction as an intriguing oncologic target. Once a subject of debate, it is now widely accepted that most cancers possess functional mitochondria, the activity of which may drive disease progression in a number of settings^25,26,33^. A recent study investigating ovarian cancer bioenergetics found that several chemoresistant cancer cell lines displayed highly upregulated mitochondrial metabolic activity as compared with their chemosensitive counterparts^27^, similar to the metabolic changes we describe in the MLM3s versus mEERLs. Interestingly, treatment with a mitochondrial inhibitor (oligomycin) significantly enhanced the cytotoxic activity of cisplatin in these lines^27^, suggesting that mitochondrial metabolism may be a particularly worthy therapeutic target in subsets of chemoresistant tumors. In HNSCC, an immunohistochemical analysis of patient samples found evidence of heightened mitochondrial respiration in proliferative tumor cells, which the authors found to be especially common in high-grade lesions^36^. A similar upregulation of mitochondrial respiration in HNSCC has been attributed to HPV positivity^37^. These findings implicate mitochondrial metabolism in the pathogenesis and progression of at least certain subsets of cancers, including HNSCC; if in part regulated by β2AR, as suggested by the data herein, this may represent a therapeutically targetable pathway.

Interestingly, propranolol’s anti-tumor activity in our HNSCC models appears to be independent of canonical β-adrenergic signaling mechanisms. Of note, β2AR is involved in a number of cAMP- and/or PKA-independent signaling pathways known to have a role in tumorigenic processes, including EGFR^38^ and PI3K transactivation^39^, calcium mobilization^40^, and Ras activation^41^, each of which may directly or indirectly modulate mitochondrial metabolism^42–46^. Future work will, therefore, aim to identify β2AR-associated signaling pathways relevant to propranolol’s activity in HNSCC. The investigation of such pathways may help to identify patient populations that would benefit from β-blockers and/or lead to novel combinatorial treatment strategies.

β-Adrenergic blockade represents a therapeutic strategy with promising translational potential. Novel anti-neoplastic drug development places an increasingly heavy burden on our society—decades-long development, soaring costs, and a low rate of FDA approval significantly limit progress in treating disease^47–49^. The pursuit of agents that are already clinically available and display oncologic potential will serve to circumvent the gridlock of novel drug development to expand clinicians’ toolboxes and provide meaningful benefit to their patients. β-blockers, given their long history of use, relative tolerability, and low cost, make for enticing—if not ideal—candidates for oncologic repurposing. Taken together, the data presented here implicate β2AR as a modulator of disease progression in HPV( + ) HNSCC and support further evaluation of this receptor as a target in the clinical management of this disease, particularly in the recurrent/metastatic setting where effective treatment strategies are notoriously lacking.

## Conclusion

β2AR contributes to an aggressive disease phenotype in a recurrent/metastatic model of HPV( + ) HNSCC and can be targeted with propranolol, a clinically available β-blocker, to inhibit primary tumor growth and metastatic development.

## Materials and methods

### Cell culture

mEERL cells were derived from C57Bl/6 murine oropharyngeal epithelial cells^50^. This HPV( + ) HNSCC model cell line is routinely grafted into syngeneic mice^30^. MLM3, derived from a spontaneously arising metastatic lung nodule in an animal implanted with a mEERL tumor and treated with standard therapies, has been previously characterized^32^. Human squamous cell carcinoma cell lines SCC1, SCC19, and SCC47 were generated at the University of Michigan (UM-SCCs) and received from Dr Douglas Trask (University of Iowa)^51^. These cell lines were recently authenticated by Genetica DNA Laboratories via DNA profiling and routinely screened by our laboratory for HPV-16 mRNA. Primary human tonsil epithelial cells were isolated from surgical tonsillectomy of consented patients under institutional IRB approval as previously described^31^ and maintained in keratinocyte serum-free medium supplemented with recombinant epidermal growth factor and bovine pituitary extract according to manufacturer protocol (Gibco). All other cell lines were maintained in Dulbecco’s modified Eagle’s medium (DMEM, Hyclone) supplemented with 10% fetal bovine serum (FBS, Atlanta Biologicals), 100 U/mL penicillin (Hyclone), and 100 μg/mL streptomycin (Hyclone). Cell lines were maintained at a humidified 37 °C in 5% CO_2_ and screened to ensure that they were free of mycoplasma.

### Western blotting

Cells were grown to 80% confluence and collected in lysis buffer (50 mM Tris HCl pH 7.5, 150 mM NaCl, 5 mM EDTA, 2 mN Na_3_VO_4_, 100 mM NaF, 10 mM NaPPi, 10% glycerol, 1% Triton X-100 [Tx-100], 17.4 μg/mL phenylmethylsulfonylfluoride, 1 × HALT with EDTA), 1% Tx-100, and HALT with EDTA (Pierce). Lysates were spun at 10,000 r.p.m. for 10 min at 4 °C. Tx-100 soluble cell lysates were subject to BCA protein assay (Pierce) and 30 μg protein/sample was separated by SDS-polyacrylamide gel electrophoresis and analyzed by western blotting with antibodies against the following targets: β2AR (Santa Cruz sc-569), α/β-Tubulin (Cell Signaling 2148 S), β-actin (Sigma-Aldrich A5316), GAPDH (ThermoFisher Scientific AM4300), and phosphorylated PKA substrate (Cell Signaling 9624). Standard horseradish peroxidase secondary antibodies (1:10,000) and ECL reagent (ThermoFisher Scientific) were used for visualization with a charge-coupled device camera imaging system (UVP). Spot densitometry was used to quantify relative expression. β2AR antibody was validated by overexpression (Supplemental Fig. 6) and CRISPR/Cas9-mediated knockout (Supplemental Fig. 5C and methods below). For validation, mouse ADRB2 cDNA (GenBank: BC032883.1) was obtained as a gBlock (IDT) and cloned into a pBabe-Zeo retroviral vector (linearized by digestion with BamHI and EcoRI) using In-Fusion cloning kit (Takara) according to manufacturer’s protocol. The resultant plasmid was sequence verified and used to transfect Phoenix cells (ATCC) via Lipofectamine 2000 (ThermoFisher Scientific) according to manufacturer’s protocol. The resultant retroviral supernatant ( + 0.4 μg/mL polybrene [Sigma]) was used to infect mEERL cells as previously described^52^.

### Cell surface immunostaining

Cells were seeded on eight-well chamber slides (Millicell EZ slide, Millipore). Before antibody incubation, slides were put on ice for 20 min. Cells were incubated with anti-β2AR antibody (Santa Cruz sc-569) at a dilution of 1:100 in phosphate-buffered saline (PBS) for 3 h. Cells were then fixed with 4% paraformaldehyde (EMD Millipore), washed with PBS, and incubated in Superblock blocking buffer (ThermoFisher Scientific) for 1 h, before incubation with Alexa-568-conjugated secondary antibody (Invitrogen) at a dilution of 1:200 in PBS for 1 h in the dark. Cells were then permeabilized with 0.2% Tx-100 (Pierce) to allow for nuclear staining and coverslips mounted with Vectashield plus DAPI (4′,6-diamidino-2-phenylindole) mounting medium (Vector Labs). Stained cells were viewed on an Olympus Fluoview 1000.

### Cellular proliferation and viability assays

For MTT proliferation assays, 5,000–10,000 cells/well were seeded (*n* = 5) in a 96-well plate in DMEM + 10% FBS and treated for 48 h with the indicated concentrations of the following compounds: atenolol (Acros Organics), ICI-118,551 (Tocris Biosciences), and propranolol (Roxane Laboratories). MTT Cell Proliferation Assay Kit (ATCC 30-1010 K) was utilized to assess proliferation according to manufacturer’s protocol. Ten microliters of MTT reagent was added to each well and cells were incubated for 4 h at 37 °C; 100 µL of detergent reagent was then added to each well and cells were incubated overnight in the dark at room temperature. Absorbance was read at 570 nm (Spectramax PLUS 384). Absorbance values of blank wells were subtracted from sample wells.

For cell counting assays, 15,000 cells/dish were seeded (*n* = 4) on 60 mm dishes in DMEM + 10% FBS. Cells were treated with 40 µM propranolol for 48 h. At 24 h time-points, cells were isolated, resuspended, and stained at a dilution of 1:1 with 0.4% trypan blue (Gibco). Live cell number and percent viability were quantified on a Countess automated hemocytometer (Invitrogen).

### Annexin V/PI apoptosis assay

A total of 250,000 cells/dish were seeded on 100 mm dishes in DMEM + 10% FBS (*n* = 4/group) and treated with 40 µM propranolol for 48 h. Culture supernatant was harvested to collect any floating dead cells and attached cells were collected via trypsinization. Cells (300,000/sample) were washed in cold PBS, before staining using fluorescein isothiocyanate (FITC) annexin V/PI kit (Invitrogen) according to manufacturer protocol. Cells were resuspended in 100 µL 1 × annexin-binding buffer (“Component C”) and incubated in FITC annexin V (“Component A”; added 3 µL/sample) and PI (“Component B”; diluted to 100 µg/mL in annexin-binding buffer; added 1 µL/sample) for 15 min. Following the incubation period, an additional 400 µL of 1 × annexin-binding buffer was added. Samples were analyzed by flow cytometry (BD Biosciences AccuriC6), measuring the fluorescence emission at 530 nm and > 575 nm. Annexin V positivity was used as a marker of apoptosis. Unstained samples were used to set the gating strategy and cells treated with 10 µM cisplatin for 48 h served as a positive control (data not shown).

### Cell cycle analysis

Cells (100,000/dish) were seeded in DMEM + 10% FBS in 60 mm dishes (*n* = 4/group) and treated with 40 µM propranolol for 48 h. Cells were collected via trypsinization and resuspended in 300 µL ice-cold PBS. Seven hundred microliters of ice-cold ethanol (final concentration = 70%) were added dropwise while vortexing. Cells were fixed for 2 h at 4 °C and washed in PBS. Cells were stained for 15 min at room temperature at a concentration of 1 × 10^6^ cells/mL in staining solution (40 µg/mL PI [Invitrogen V13241], 50 µL/mL RNase cocktail enzyme mix [Invitrogen AM2286], 0.1% Tx-100 in PBS). Samples were analyzed by flow cytometry (BD Biosciences AccuriC6). Data were analyzed using ModFit LT software package (http://www.vsh.com/products/mflt/index.asp).

### Colony-forming assay

Four hundred cells/well were seeded in a 12-well plate (*n* = 4/group) and treated with the indicated concentration of propranolol. Once control group colonies reached a sufficient size (~96 h), cells were washed with PBS, fixed with 70% ethanol for 5 min, stained with crystal violet (0.5% crystal violet w/v, 10% ethanol), washed twice with PBS, and allowed to air dry. Samples were analyzed using the GelCount system and associated software (Oxford Optronix).

### Scratch wound migration assay

Cells (50,000/well) were seeded in DMEM + 10% FBS on collagen-coated 12-well plates and allowed to grow to form a confluent monolayer. Once confluent, cells were serum starved (DMEM + 0% FBS) for 8 h to prevent confounding effects of treatment on proliferation (although it is also worth noting that significant differences in cellular proliferation were not observed within 24 h of treatment [Fig. 3a, b]). A P200 pipette tip was used to generate uniform wounds in the shape of crosshairs. Cells were rinsed in DMEM to remove wound debris and treated with propranolol (40 µM in DMEM + 0.5% FBS) overnight. Bright-field images (EVOS xl) were taken of each well at *t* = 0 h and *t* = 16 h. TScratch (http://www.cse-lab.ethz.ch/) software, developed by the Koumoutsakos and colleagues^53^ (CSE Lab), at ETH Zurich, was used to quantify percent wound closure.

### Phalloidin staining

Ten thousand cells/well were seeded on eight-well chamber slides and treated with propranolol for 16 h. Cells were washed twice with PBS, fixed with 4% paraformaldehyde (EMD Millipore), washed twice with PBS, permeabilized with 0.2% Tx-100 in PBS, and incubated with AlexaFluor 488 phalloidin (Life Technologies A12379) diluted 1:50 in Superblock blocking buffer (ThermoFisher Scientific) for 2 h at 37 °C in the dark. Cells were washed three more times with PBS and coverslips were mounted with Vectashield plus DAPI mounting medium (Vector Labs). Stained cells were viewed on an Olympus Fluoview 1000.

### **Seahorse mitochondrial stress test**

OCR was measured on a Seahorse XF24 extracellular flux analyzer (Agilent) according to manufacturer protocol for mitochondrial stress testing. Briefly, 25,000 cells/well were seeded on a Seahorse XF24 Cell Culture Microplate (Agilent) in DMEM + 10% overnight, followed by treatment with 40 µM propranolol for 24 h. Before running the assay, medium was changed to fresh, pre-warmed mitochondrial stress test medium (XF Base Medium [Agilent], 1 mM sodium pyruvate [Fisher], 2 mM glutamine [Sigma-Aldrich], and 10 mM glucose [Sigma-Aldrich], pH 7.4, sterile-filtered) and cells were incubated at 37 °C in a non-CO_2_ incubator for 1 h. Mitochondrial stress test reagents (Agilent) were diluted in mitochondrial stress test medium and loaded into individual ports of a Seahorse XF24 Sensor Cartridge (Agilent) that had been hydrated in Seahorse XF Calibrant Solution (Agilent) overnight at 37 °C. Final concentrations of stress test reagents were as follows: 1 µM oligomycin (oligo), 2 µM FCCP, 0.5 µM rot/AA. Instrument protocol commands were as follows: calibrate; equilibrate; loop 3 × : 3 min mix, 2 min wait, 3 min measure; inject port A; repeat loop for each injected compound. Sample readings were adjusted for non-mitochondrial respiration (OCR following rot/AA injection). Basal OCR reflects average OCR prior to oligomycin injection; ATP-linked OCR reflects the difference between basal OCR and OCR following oligomycin injection; maximal OCR reflects max OCR following FCCP injection. Data represent non-mitochondrial respiration-adjusted OCR normalized to total protein (quantified per well via BCA protein assay [Pierce]), depicted as percent relative to control basal OCR.

### **CRISPR/Cas9-mediated generation and screening of β2AR-null clones**

Two distinct locations within the mouse β2AR gene sequence were targeted separately to induce a premature termination codon via insertion/deletion-mediated frameshift mutations. Target selection and guide sequence cloning were carried out using the tools and protocol described by Ran et al.^54^. Guides targeted the following sequences, located on the anti-sense strand (PAM sequence in bold): 5′-cccgttcctgagtgacgtcg**TGG** -3′; 5′-aggacgcgatggcgtaggcc**TGG**-3′. Target sequences were selected based on restriction enzyme sites to allow for screening of mutants via restriction fragment length polymorphism (RFLP). The PCR product of a restriction enzyme-digested wild-type sequence will display two bands on gel electrophoresis; a bi-allelic mutant will display one band. PCR primers used for RFLP-based clone screening are listed in Table 1, along with product size. Restriction digest with DrdI or StuI was used to screen for early exon target and middle exon target mutations, respectively (Supplemental Fig. 2A and 2B). Positive clones were confirmed for loss of protein via western blotting (Supplemental Fig. 2C). One clone from each targeting strategy was selected for experimentation.

**Table 1:** PCR primer sequences and corresponding product sizes for clone screening via RFLP

Primer	Sequence (5′– 3′)	Amplicon (bp)	Cut product (bp)	Enzyme
Early Exon FWD	AATGAAGCTTCCAGGAGTCCG	664	488 and 178	DrdI
Early Exon REV	AACAATCGATAGCTTTCTTGTGGG			
Mid Exon FWD	ATCCTCATGTCGGTTATCGTCC	627	481 and 146	StuI
Mid Exon REV	GCTGAGGTTTTGGGCGTGG			

### Animal studies

In vivo models of HPV( + ) HNSCC were utilized as described previously^30^. All experiments were performed in accordance with institutional and national guidelines, and were approved by the Institutional Animal Care and Use Committee at Sanford Research. Briefly, 50,000 cells were injected in a volume of 100 µL DMEM subcutaneously (day 0) into the hind limb of male C57Bl/6 (Jackson Laboratories). Mice weighed 20–25 g and were 4–8 weeks old. Groups were assigned arbitrarily. Mice were treated daily with 2 mg/ kg IP propranolol (Sigma-Aldrich) or vehicle (bacteriostatic 0.9% sodium chloride [Hospira, Inc.] and 5% ethanol). CRT began at day 7 and mice were treated once per week for 3 weeks with 0.132 mg/mouse (20 mg/m^2^) IP cisplatin (Calbiochem) dissolved in bacteriostatic 0.9% sodium chloride (Hospira Inc.), and 8 Gy (24 Gy total [RS2000 irradiator, RadSource Technologies, Inc. Suwanee, GA]), respectively, under ketamine/xylazine anesthesia (87.5 mg/kg ketamine; 12.5 mg/kg xylazine). Cisplatin dose is based on equivalent human dosing^30^. Tumor volume was measured weekly as previously described^50^. During measurement, animals were identified only by cage number and animal number (ear punch). Animals were euthanized when tumor volume was > 1.5 cm in any dimension, or when animals displayed significant edema, ulceration, lameness, or cachexia. Survival statistics were calculated by standardizing for a tumor volume of 2500 mm^3^.

### **Lung metastasis immunohistochemistry**

Tumor cells were implanted and mice treated as described above. After 3 weeks, mice were killed and lungs were fixed in 10% buffered formalin, paraffin embedded, and stained for anti-cytokeratin (1:100, ab9377, Abcam). Tissues were sectioned to 5 μm. A BenchMark® XT automated slide staining system (Ventana Medical Systems, Inc.) was used for staining with antibodies as per standard protocols. Three random, independent lung sections from each animal were stained to ensure a global representation of metastasis within the lungs. Slides were identified solely by cage number and animal number. Lung sections were manually scanned on an Olympus DP71 microscope and the number of large ( > 70 μm diameter) and small metastatic lung nodules counted.

### Statistical analysis

SigmaPlot 14.0 (Systat Software, Inc.) and Excel 2016 (Microsoft) were used to conduct statistical analyses. Mann–Whitney test was used to compare two groups with small sample size (*n* < 10) or non-normally distributed data (*p* < 0.05 on Shapiro–Wilk normality test), and two-tailed, unpaired, Student’s *t*-test was used to compare groups with large sample size (*n* > 10), unless otherwise noted. Brown–Forsythe test was used to test for equal variance (*p* > 0.05) before running student’s *t*-test. Log-rank test was used to compare differences in survival. *χ*^2^-test was used to compare proportions of metastasis-positive mice. *p*-Values ≤ 0.05 were considered significant. Sample sizes for animal studies were estimated based on an alpha of 0.05 and power of 0.80. The propranolol study was set to detect a difference in means of 800 mm^3^, propranolol + CRT study: 500 mm^3^, and CRISPR study: 1000 mm^3^. Estimated SDs were set to 500 mm^3^.

## Electronic supplementary material

Supplemental Figures
