# Supplementary material for: β2-Adrenergic receptor modulates mitochondrial metabolism and disease progression in recurrent/metastatic HPV(+) HNSCC
Source: Oncogenesis. 2018 Oct 8;7(10):81. doi: 10.1038/s41389-018-0090-2 (PMC6175933; doi:10.1038/s41389-018-0090-2)
Supplement: Supplementary file 1 — Supplemental Figures [file 41389_2018_90_MOESM1_ESM.pptx]

## Slide 1
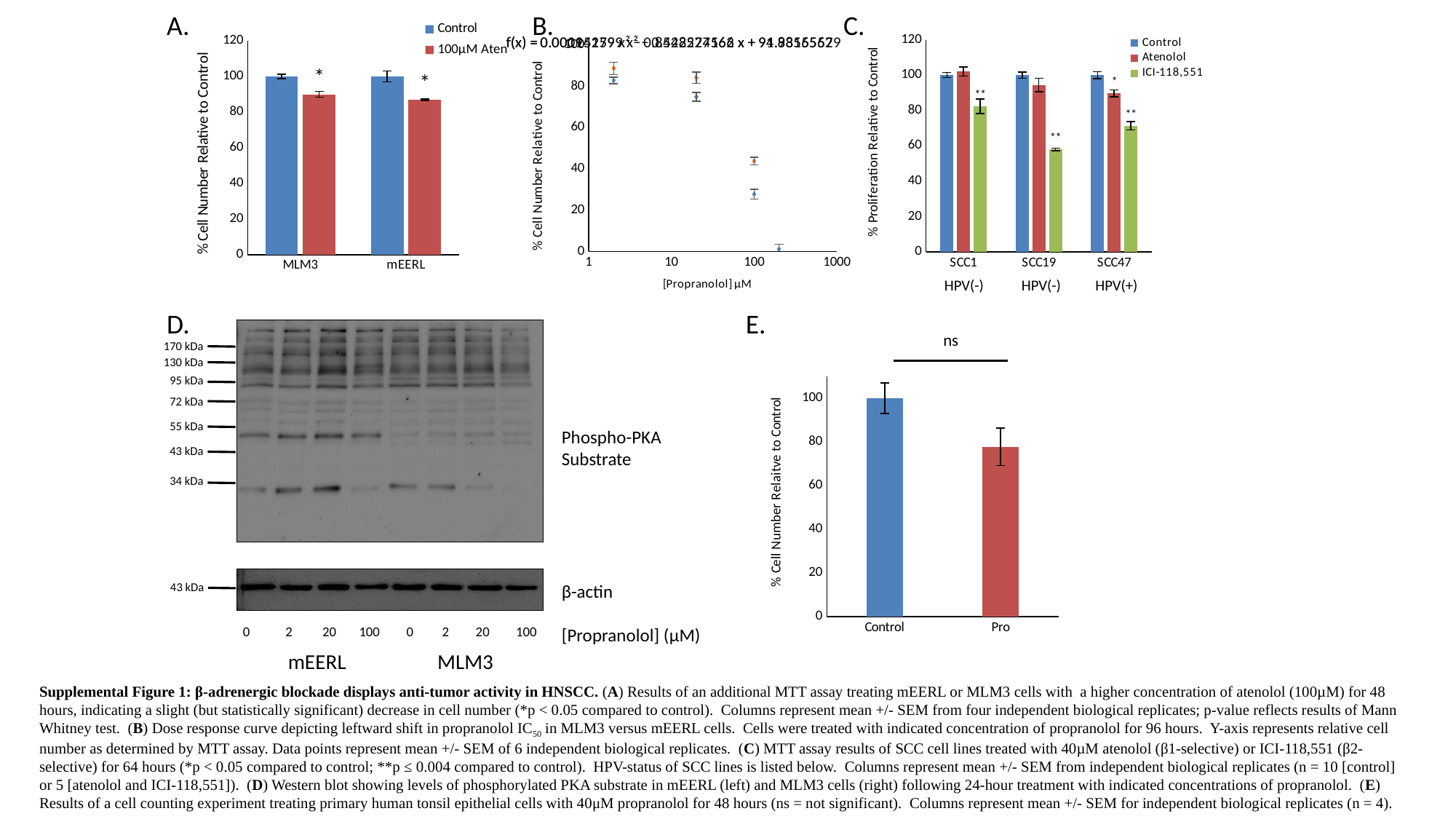

A.
B.
C.
### Chart
| Category | Control | 100μM Aten |
|---|---|---|
| MLM3 | 100.00000000000001 | 89.94658119658119 |
| mEERL | 100.0 | 86.93470838269111 |
### Chart
| Category | Control | | |
|---|---|---|---|
| SCC1 | 99.99999999999999 | 101.92754248607773 | 82.31099603394776 |
| SCC19 | 100.00000000000003 | 94.38391946992867 | 57.91762232415903 |
| SCC47 | 100.00000000000003 | 89.62798580484473 | 71.33346442315795 |
### Chart
| Category | | |
|---|---|---|| HPV(-) | HPV(-) | HPV(+) |
| --- | --- | --- |
### Chart
| Category | |
|---|---|
| Control | 100.00000000000001 |
| Pro | 77.77777777777777 |ns
D.
E.
170 kDa
130 kDa
95 kDa
72 kDa
55 kDa
Phospho-PKA
Substrate
43 kDa
34 kDa
43 kDa
β-actin
[Propranolol] (μM)
| 0 | 2 | 20 | 100 | 0 | 2 | 20 | 100 |
| --- | --- | --- | --- | --- | --- | --- | --- |
mEERL
MLM3
Supplemental Figure 1: β-adrenergic blockade displays anti-tumor activity in HNSCC. (A) Results of an additional MTT assay treating mEERL or MLM3 cells with a higher concentration of atenolol (100µM) for 48 hours, indicating a slight (but statistically significant) decrease in cell number (*p < 0.05 compared to control). Columns represent mean +/- SEM from four independent biological replicates; p-value reflects results of Mann Whitney test. (B) Dose response curve depicting leftward shift in propranolol IC50 in MLM3 versus mEERL cells. Cells were treated with indicated concentration of propranolol for 96 hours. Y-axis represents relative cell number as determined by MTT assay. Data points represent mean +/- SEM of 6 independent biological replicates. (C) MTT assay results of SCC cell lines treated with 40µM atenolol (β1-selective) or ICI-118,551 (β2-selective) for 64 hours (*p < 0.05 compared to control; **p ≤ 0.004 compared to control). HPV-status of SCC lines is listed below. Columns represent mean +/- SEM from independent biological replicates (n = 10 [control] or 5 [atenolol and ICI-118,551]). (D) Western blot showing levels of phosphorylated PKA substrate in mEERL (left) and MLM3 cells (right) following 24-hour treatment with indicated concentrations of propranolol. (E) Results of a cell counting experiment treating primary human tonsil epithelial cells with 40μM propranolol for 48 hours (ns = not significant). Columns represent mean +/- SEM for independent biological replicates (n = 4).

## Slide 2
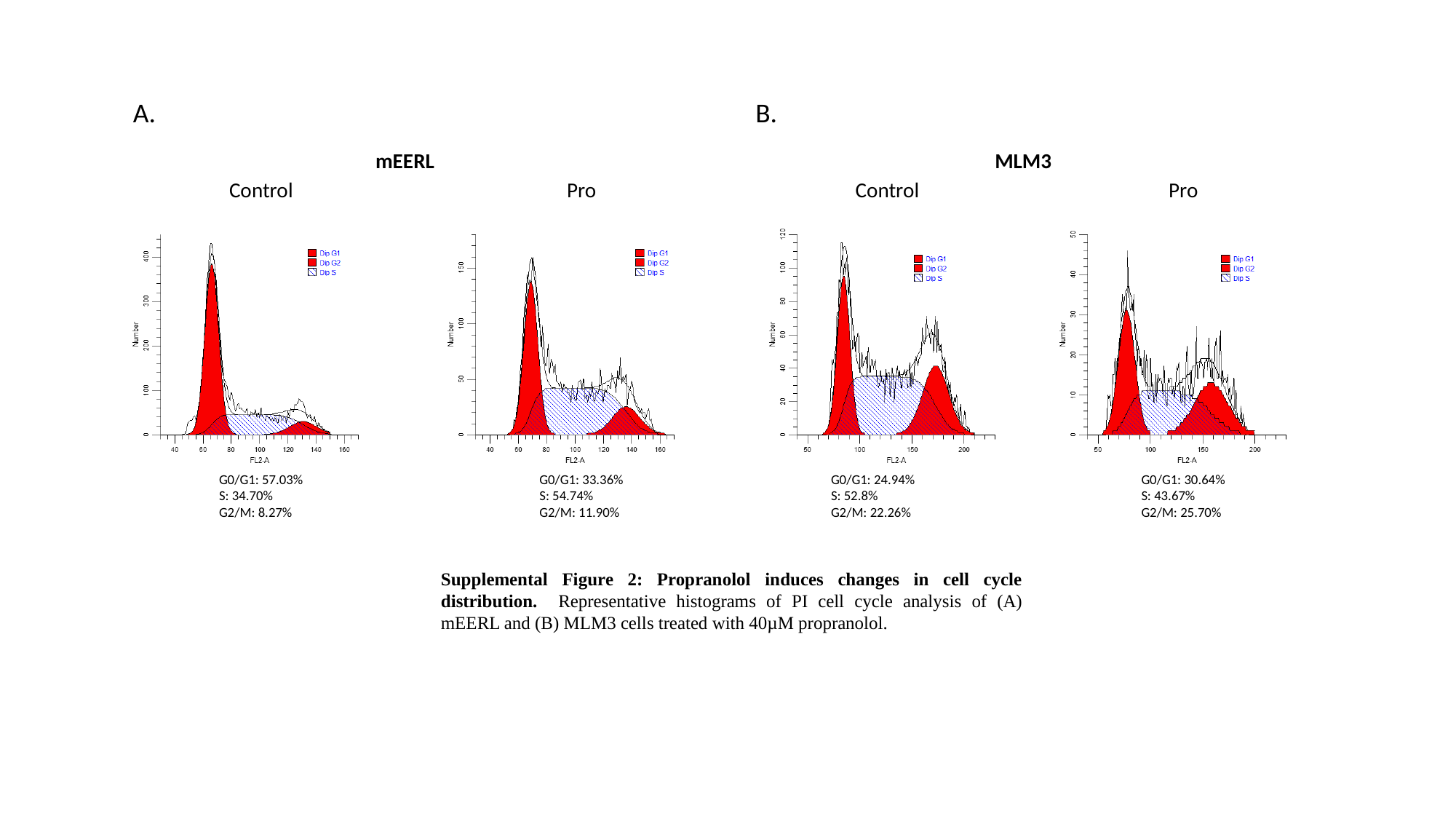

A.
B.
mEERL
MLM3
Control
Pro
G0/G1: 57.03%
S: 34.70%
G2/M: 8.27%
G0/G1: 33.36%
S: 54.74%
G2/M: 11.90%
Control
Pro
G0/G1: 24.94%
S: 52.8%
G2/M: 22.26%
G0/G1: 30.64%
S: 43.67%
G2/M: 25.70%
Supplemental Figure 2: Propranolol induces changes in cell cycle distribution. Representative histograms of PI cell cycle analysis of (A) mEERL and (B) MLM3 cells treated with 40µM propranolol.

## Slide 3
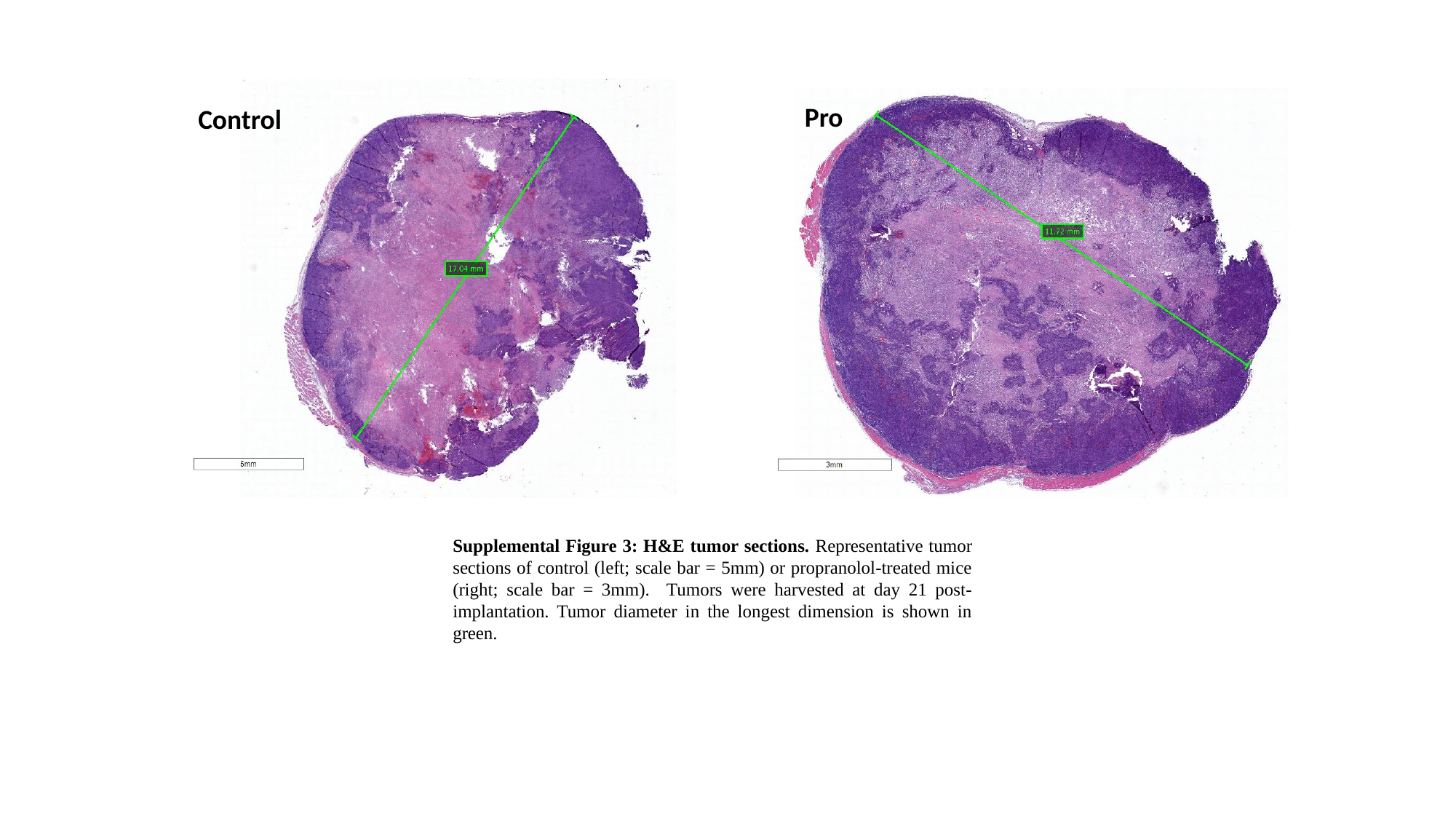

Pro
Control
Supplemental Figure 3: H&E tumor sections. Representative tumor sections of control (left; scale bar = 5mm) or propranolol-treated mice (right; scale bar = 3mm). Tumors were harvested at day 21 post-implantation. Tumor diameter in the longest dimension is shown in green.

## Slide 4
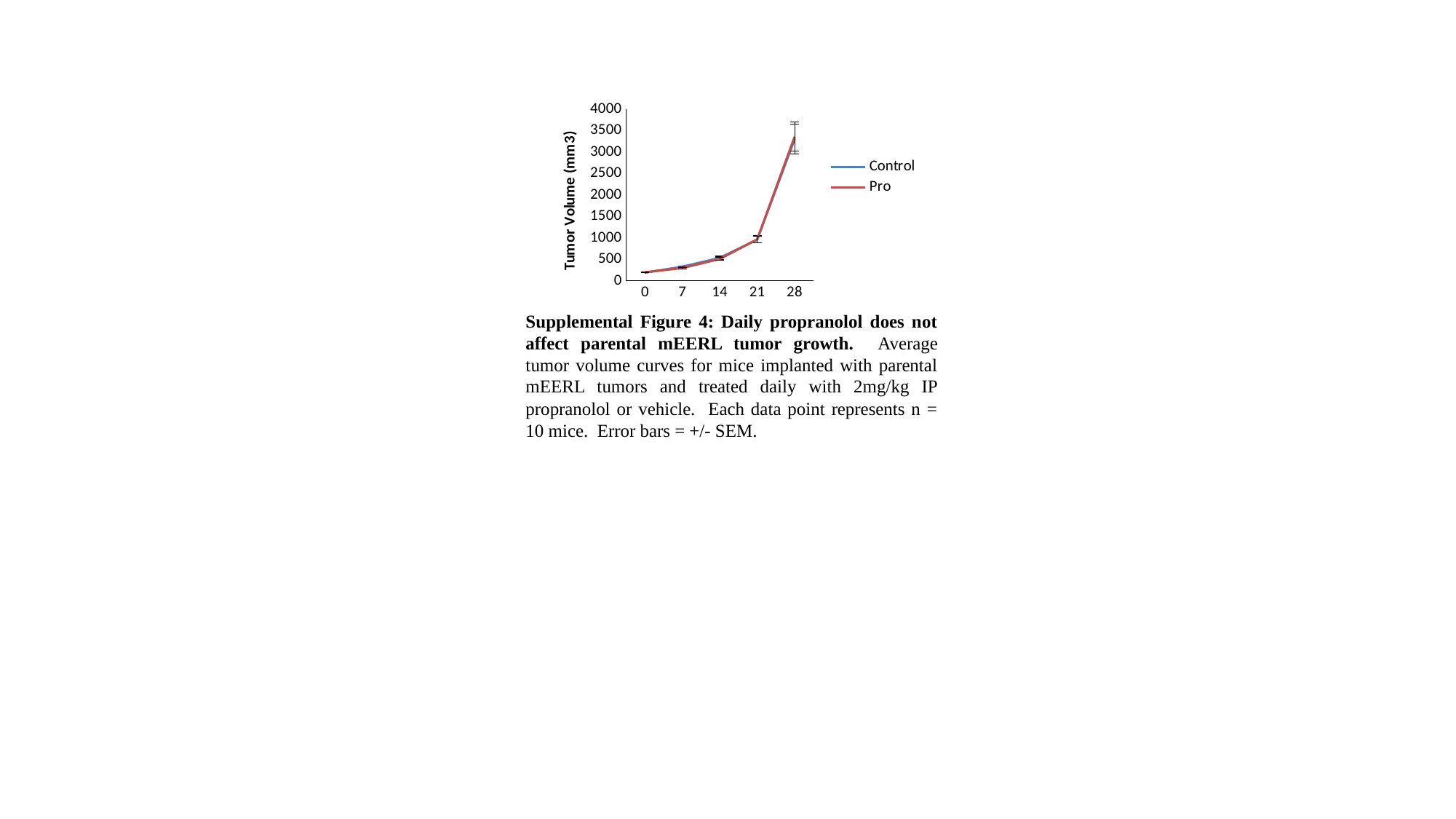

### Chart
| Category | | |
|---|---|---|
| 0 | 192.20355555555557 | 200.57799999999997 |
| 7 | 331.8496666666666 | 300.11129999999997 |
| 14 | 536.4091111111112 | 511.3336000000001 |
| 21 | 961.3529333333333 | 967.1636732999999 |
| 28 | 3302.4494444444445 | 3355.2952000000005 |Supplemental Figure 4: Daily propranolol does not affect parental mEERL tumor growth. Average tumor volume curves for mice implanted with parental mEERL tumors and treated daily with 2mg/kg IP propranolol or vehicle. Each data point represents n = 10 mice. Error bars = +/- SEM.

## Slide 5
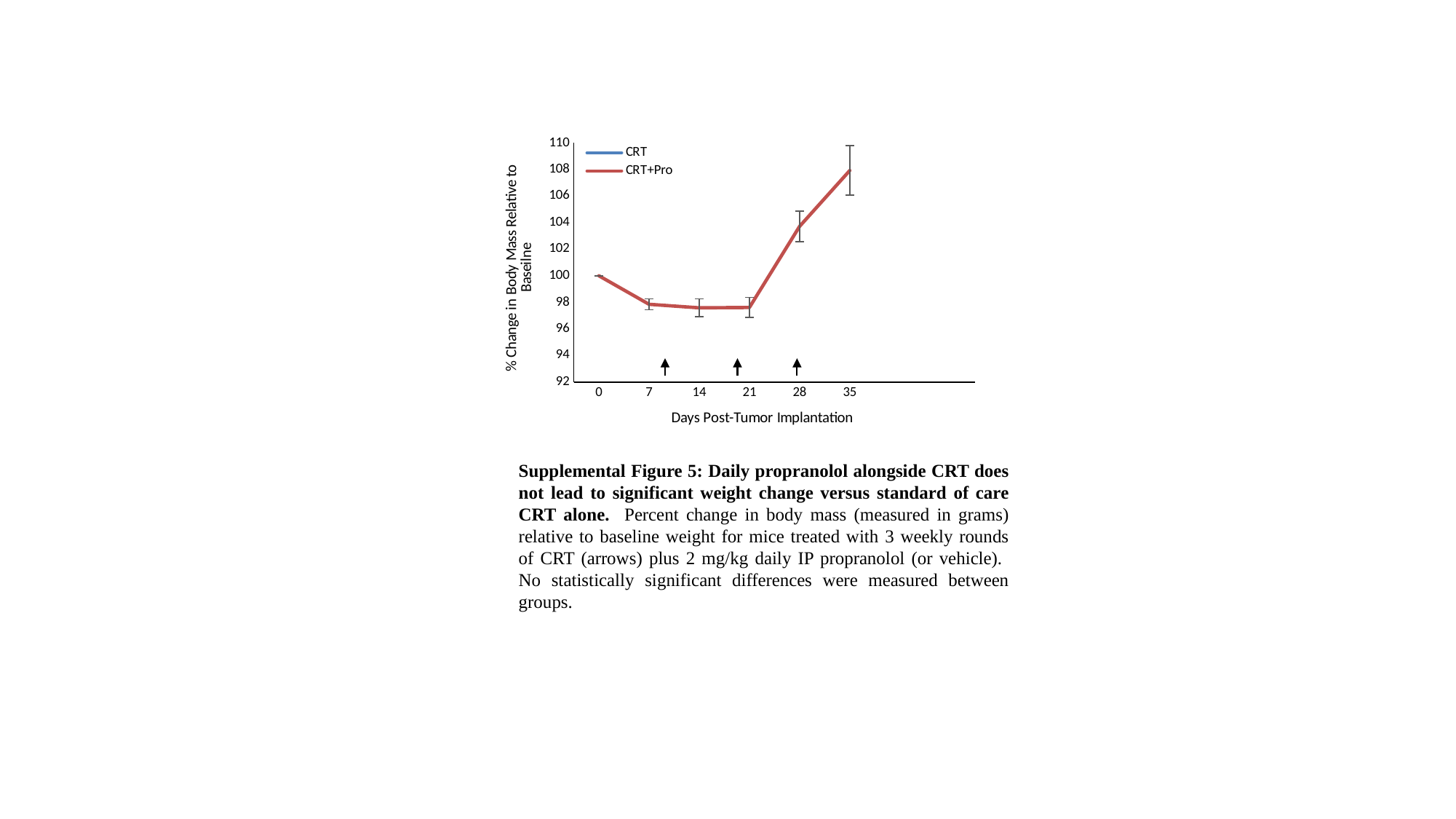

### Chart
| Category | | |
|---|---|---|
| 0 | 100.0 | 100.0 |
| 7 | 98.25835685948557 | 97.84722811323668 |
| 14 | 96.40723641269072 | 97.58463201220907 |
| 21 | 96.39851856249555 | 97.60794622083787 |
| 28 | 101.65157849167893 | 103.71813518490632 |
| 35 | 105.02792795674027 | 107.92161143707973 |Supplemental Figure 5: Daily propranolol alongside CRT does not lead to significant weight change versus standard of care CRT alone. Percent change in body mass (measured in grams) relative to baseline weight for mice treated with 3 weekly rounds of CRT (arrows) plus 2 mg/kg daily IP propranolol (or vehicle). No statistically significant differences were measured between groups.

## Slide 6
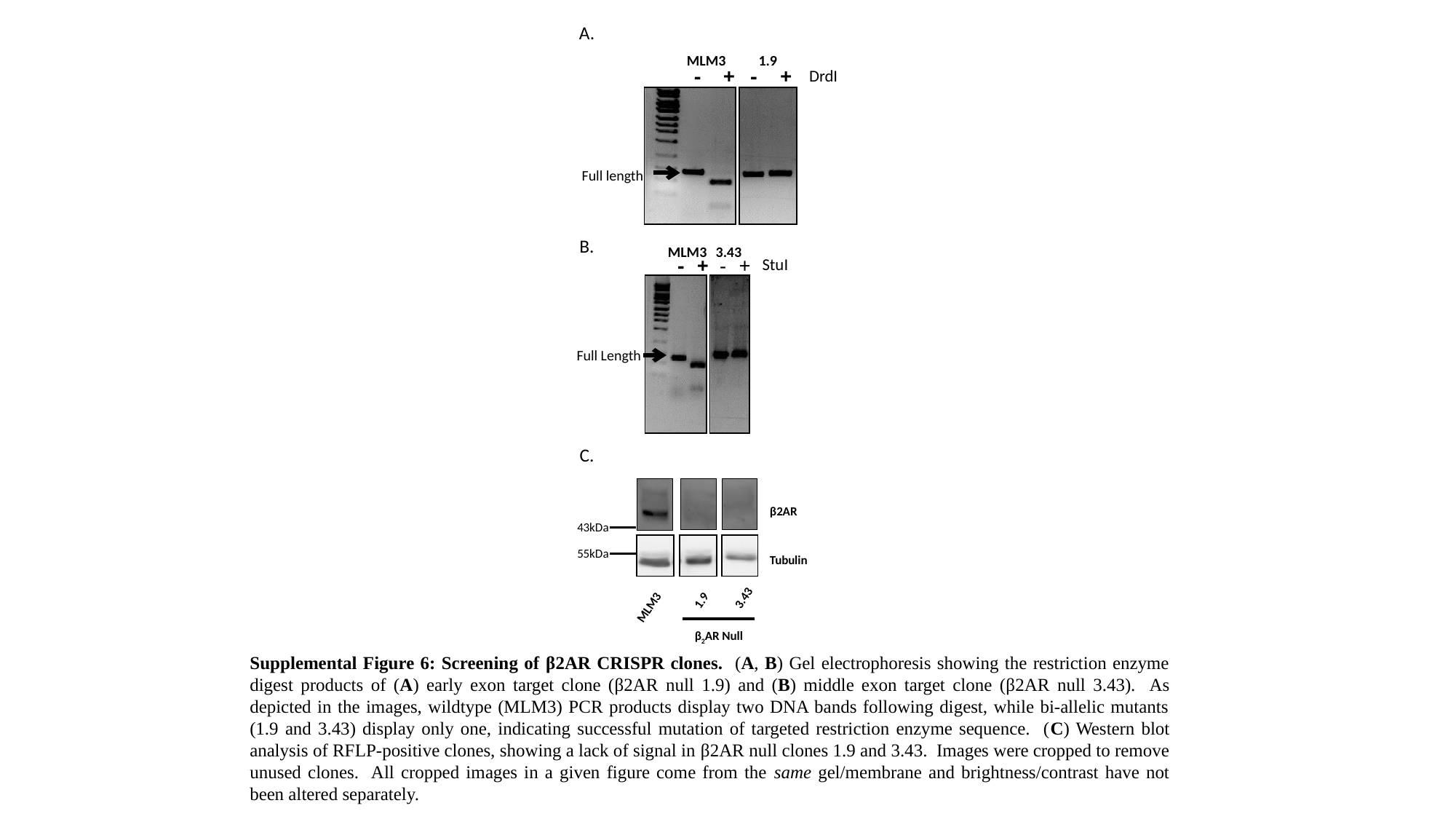

A.
MLM3
1.9
-
+
-
+
DrdI
Full length
B.
MLM3
3.43
-
+
-
+
StuI
Full Length
C.
β2AR
43kDa
55kDa
Tubulin
3.43
1.9
MLM3
β2AR Null
Supplemental Figure 6: Screening of β2AR CRISPR clones. (A, B) Gel electrophoresis showing the restriction enzyme digest products of (A) early exon target clone (β2AR null 1.9) and (B) middle exon target clone (β2AR null 3.43). As depicted in the images, wildtype (MLM3) PCR products display two DNA bands following digest, while bi-allelic mutants (1.9 and 3.43) display only one, indicating successful mutation of targeted restriction enzyme sequence. (C) Western blot analysis of RFLP-positive clones, showing a lack of signal in β2AR null clones 1.9 and 3.43. Images were cropped to remove unused clones. All cropped images in a given figure come from the same gel/membrane and brightness/contrast have not been altered separately.

## Slide 7
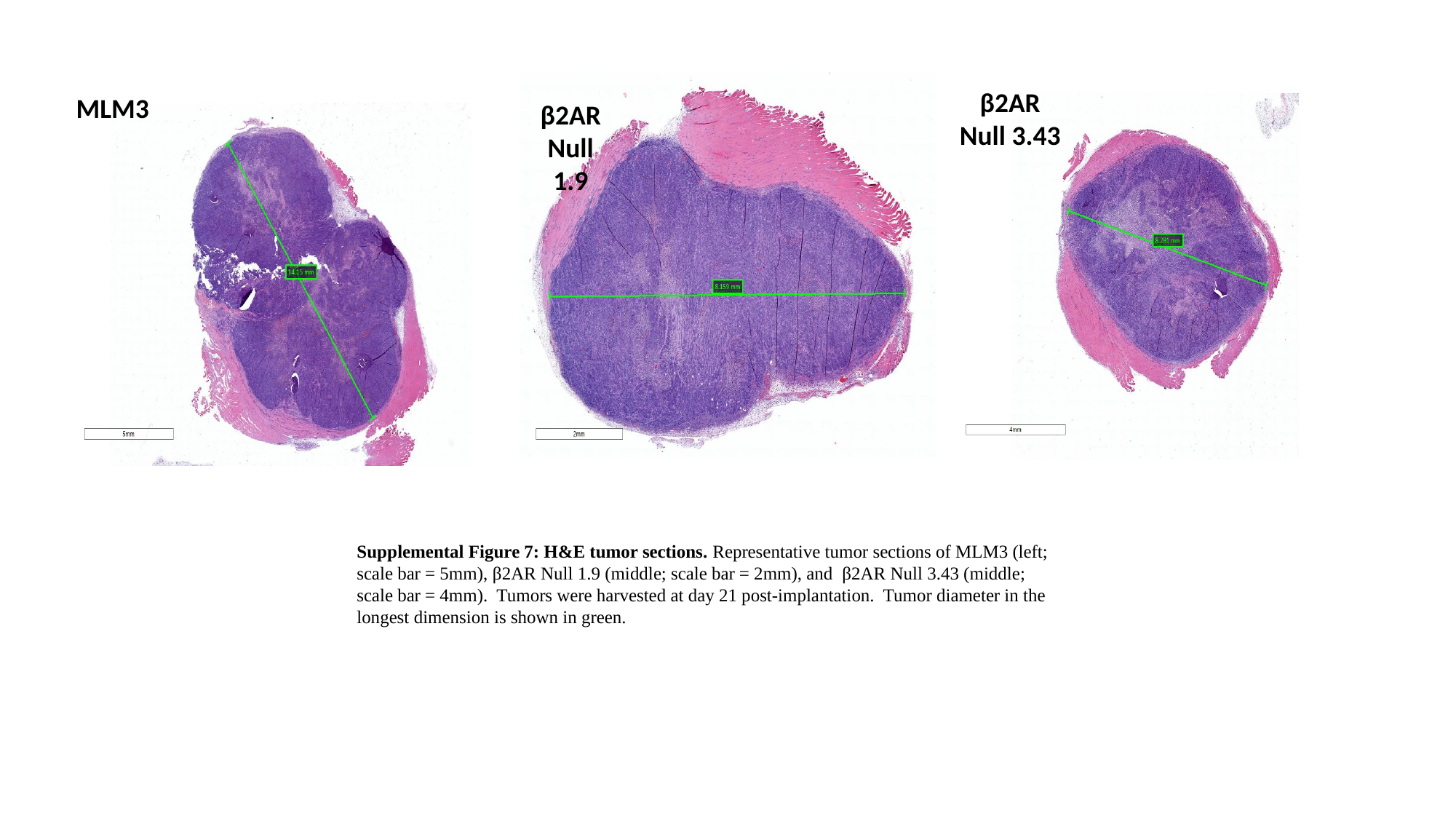

MLM3
β2AR Null
1.9
β2AR Null 3.43
Supplemental Figure 7: H&E tumor sections. Representative tumor sections of MLM3 (left; scale bar = 5mm), β2AR Null 1.9 (middle; scale bar = 2mm), and β2AR Null 3.43 (middle; scale bar = 4mm). Tumors were harvested at day 21 post-implantation. Tumor diameter in the longest dimension is shown in green.

## Slide 8
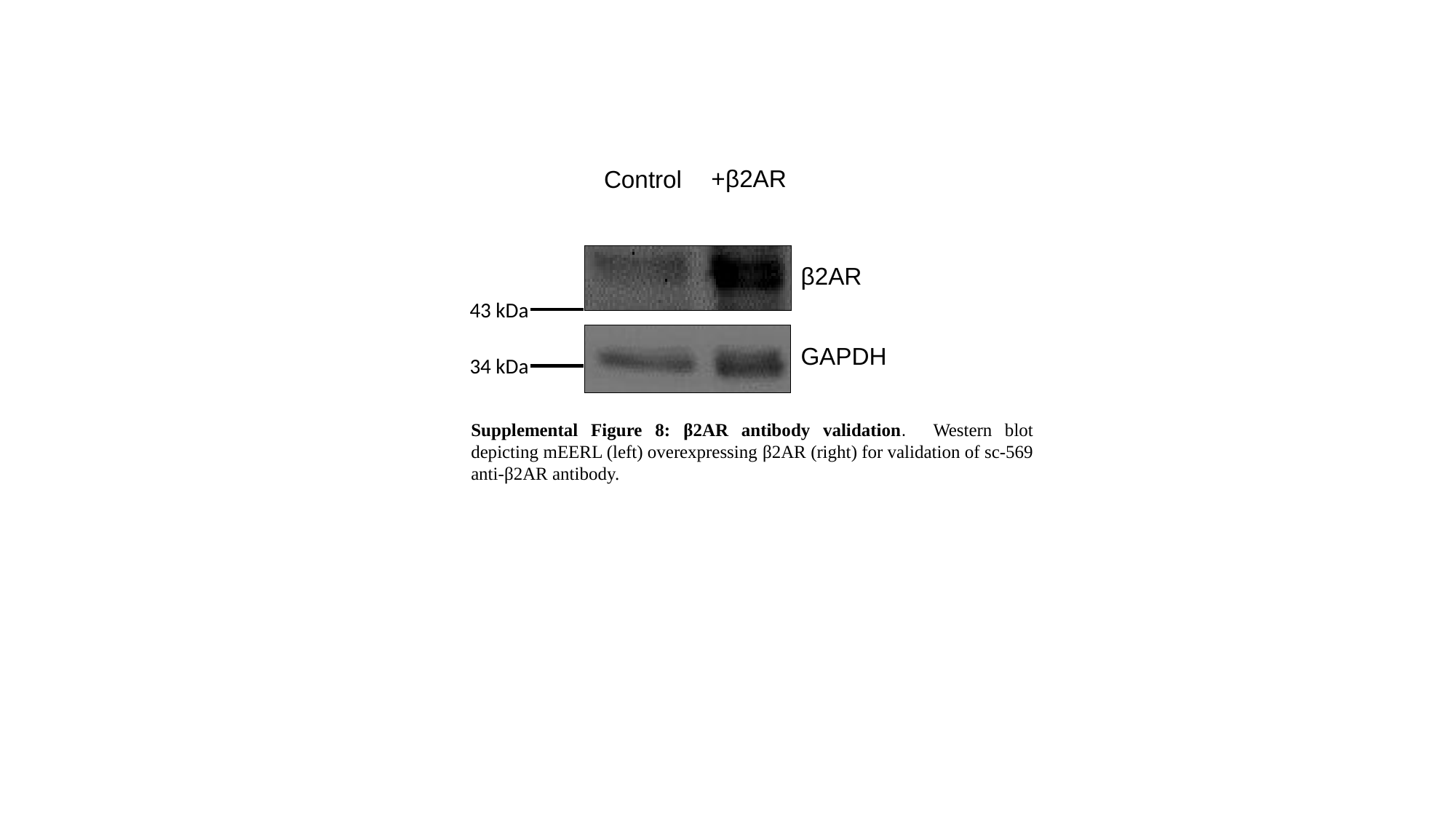

+β2AR
Control
β2AR
43 kDa
GAPDH
34 kDa
Supplemental Figure 8: β2AR antibody validation. Western blot depicting mEERL (left) overexpressing β2AR (right) for validation of sc-569 anti-β2AR antibody.
